# Genome Wide Association Studies with Different Weighting Approaches Reveals Genomic Windows Associated with Meat Quality Traits in Beef Cattle

**DOI:** 10.3390/genes17040385

**Published:** 2026-03-28

**Authors:** Hugo Borges Dos Reis, Amanda Marchi Maiorano, Elisângela Oliveira, Filippi Tonetto, Fernando Baldi, Breno de Oliveira Fragomeni, José Bento Sterman Ferraz

**Affiliations:** 1 Faculdade de Zootecnia e Engenharia de Alimentos, Universidade de São Paulo, Pirassununga 13635-900, SP, Brazil; hugoreis@usp.br (H.B.D.R.); jbferraz@usp.br (J.B.S.F.); 2 Faculdade de Medicina Veterinária e Zootecnia, Universidade Federal de Uberlândia, Uberlandia 38400-902, MG, Brazil; 3 Fazenda Perfeita União, Bauru 16600-000, SP, Brazil; 4 Department of Animal Sciences, University of Connecticut, Storrs, CT 06269, USA

**Keywords:** genetic architecture, zebu breed, carcass quality

## Abstract

**Background/Objectives:** Genome-wide association studies (GWAS) based on single-step genomic BLUP (ssGBLUP) commonly assume equal single nucleotide polymorphism (SNP) variances, which may not reflect the biological architecture of complex traits. Alternative weighting strategies can increase detection power but may affect stability. This study evaluated how different SNP weighting approaches influence genomic region detection and biological interpretation of ribeye area (REA) and subcutaneous fat thickness (SFT) in Guzerá cattle. **Methods:** Phenotypic records from 2729 animals and genotypes from 1405 individuals (43,039 SNPs after quality control) were analyzed. Heritabilities were estimated using Restricted Maximum Likelihood (REML), and GWAS were conducted under five approaches: unweighted method (UM), quadratic method (QM), and three Non-Linear A strategies with weighting constants (1.125, 1.2, and 1.5). Genomic windows of 20 adjacent SNPs explaining ≥0.5% of the additive genetic variance (AGV) were considered significant. Recurrent regions were prioritized, and functional enrichment analyses (KEGG, GO, and MeSH) were performed. **Results:** Heritability estimates were moderate for REA (0.26 ± 0.05) and SFT (0.22 ± 0.04). Weighted approaches increased detection sensitivity. For REA, UM identified 10 windows, whereas QM and A_1.5 detected 24 and 31 windows. For SFT, UM identified 8 windows, while QM and A_1.5 detected 30 and 23 windows. Recurrent chromosomes included 2, 4, 6, 12, 16, 19, and 22 for REA, and 2, 3, 5, 7, 11, 17, and 22 for SFT. Key genes included *AKT3*, *NOS2*, and *MSTN*. Enrichment highlighted pathways related to muscle growth and lipid metabolism. **Conclusions:** SNP-weighted GWAS increased detection sensitivity but involved trade-offs between signal amplification and stability. Integrating weighting strategies improves biological interpretation and supports robust candidate gene identification for genomic selection.

## 1. Introduction

The use of SNP markers in genomic selection models has revolutionized animal breeding by providing a precise, stable, and widely distributed way to capture genetic variation across the genome [1]. Because SNPs are expected to be in linkage disequilibrium (LD) with functional variants, they enable tracking of genomic regions associated with phenotypes of interest, thereby increasing the accuracy of genetic estimates, especially for complex traits such as REA and SFT.

These traits are widely used in beef cattle breeding programs because they are positively correlated with carcass weight and animal growth [2,3,4]. Given the importance of these traits for production efficiency, GWAS have been widely employed to investigate the underlying genetic architecture and to identify genomic regions associated with their expression. GWAS results contribute to the understanding of the genetic mechanisms involved and provide support for the improvement of genomic models and the biological interpretation of phenotypes.

The GWAS is based on sequentially testing each SNP to determine its association with the trait under study, assuming that any significant association results from the SNP being in LD with a nearby causal mutation that actually affects the phenotype [5]. The SNP effect or variance can be derived from the Genomic Best Linear Unbiased Prediction (GBLUP) method, which assumes a normal distribution for SNP effects and calculates them from phenotypes and a genomic relationship matrix [6].

When part of the population is genotyped, ssGBLUP is used as a common and flexible method for genomic prediction that allows the simultaneous incorporation of genomic, phenotypic, and pedigree information in a single system of equations, increasing the accuracy and consistency of estimates [7,8]. The GBLUP and ssGBLUP methods usually assume equal weights for all SNPs [6]. However, this assumption is biologically incorrect, and to overcome this limitation, ref. [9] proposed an alternative method by modifying ssGBLUP to establish a way to calculate and assign different weights to each SNP. This approach was named weighted ssGBLUP (WssGBLUP), in which the weights assigned to SNPs are derived from the square of their respective solutions.

However, some of these methods, when used iteratively, diverge because initially large effects become very large, and effects that are a priori small become very small or null [10]. An alternative would be to use the Nonlinear-A weighting methodology, which can reduce abrupt changes in SNP weights across iterations, minimizing potential inflation of the results. In this way, studies have been developed to investigate alternative SNP weighting approaches [11,12,13]. The ideal weighting strategy depends on the trait’s genetic architecture and data quality [12]. Additionally, using incorrect weighting approaches may lead to spurious or false negative associations.

Although SNP weighting approaches have been widely explored for improving genomic prediction accuracy, comparatively few studies have systematically evaluated how different weighting intensities influence the detection of genomic regions and biological interpretation of complex carcass traits, particularly in zebu beef cattle populations characterized by extended linkage disequilibrium. Therefore, understanding how distinct SNP weighting strategies affect sensitivity, stability, and biological consistency of GWAS results is crucial to avoid inflated signals and improve the reliability of candidate gene identification.

The objective of this study was to evaluate how different SNP weighting strategies affect the detection of genomic regions associated with carcass traits in beef cattle, as well as to assess the recurrence of these regions across approaches and the biological consistency of candidate genes and enriched pathways for REA and SFT.

## 2. Materials and Methods

### 2.1. Animal Care and Use

Institutional Animal Care and Use Committee (IACUC) approval was not required for this study because all data were obtained from an existing database and did not involve any new animal procedures or interventions.

### 2.2. Animals, Genotyping, and Quality Control

Phenotypic data for Guzerá cattle were provided by the National Association of Breeders and Researchers (ANCP), and the carcass traits were evaluated by a technician from Aval – Technological Services (Piracicaba, SP, Brazil), accredited by the Ultrasound Guidelines Council (UGC, Pleasantville, IA, USA). The traits analyzed were ribeye area (REA) and subcutaneous fat thickness (SFT), measured by real-time ultrasonography. Data were restricted to phenotypes recorded between 2012 and 2024. A total of 2729 animals with phenotypic information were included in the dataset, comprising 1989 males and 740 females. The number of observations varied by trait, with 2697 records available for REA and 2141 for SFT, as not all animals were measured for both phenotypes. Animals had an average age of 517 ± 49 days and a mean body weight of 440 ± 85 kg at measurement. All animals were raised under similar management conditions in pasture-based production systems, receiving mineral supplementation and water ad libitum.

The pedigree dataset included 11,514 records, and a total of 1916 animals were genotyped using the GeneSeek Genomic Profiler (GGP) Bovine 50K SNP panel (Neogen Corporation, Lincoln, NE, USA), comprising 53,492 markers over the 29 autosomes and the X chromosome. For each sample, overall genotyping quality control was evaluated by assessing the genotype call rate, defined as the ratio between the number of called genotypes and the total number of markers. Samples with a call rate lower than 0.9 (<90%) were discarded. Mendelian inconsistencies were also assessed, and animals with conflicting genotypes were removed. For the GWAS, SNP quality control involved the removal of monomorphic markers and SNPs with a minor allele frequency (MAF) below 0.05. Call rate was further verified, and SNPs with rates below 0.90 were excluded. Additionally, SNPs showing Mendelian conflicts in parent-offspring relationships were removed. SNP positions and chromosomal coordinates were based on the bovine reference genome assembly ARS-UCD 2.0. Quality control steps were performed using the preGSf90 program [14]. As a result, a final dataset comprising 43,039 SNPs and 1405 genotyped animals was obtained and used for subsequent analyses.

### 2.3. Model

A single-trait animal model was fitted for both traits. The fixed effects included contemporary group (CG) and age at measurement (in days), fitted as a linear covariate. The direct additive genetic effect of the animal and the residual associated with each observation were included as random effects. CGs were defined based on the combination of sex, management system, year of birth, and season of birth and comprised 64 levels. Groups containing fewer than three observations, as well as those with outlying values (±3 standard deviations from the group mean), were excluded from the analysis. Genetic parameters and variance components were estimated using an average information REML algorithm [15]. The estimation of variance components and heritability was performed to obtain the AGV required for subsequent GWAS analyses.

The statistical model can be represented in matrix form as follows:

(1) y=Xβ+Za+e

where ***y*** represents the vector of observations, **β** is the vector of fixed effects; where **a** is the vector of direct additive genetic effects; **X** is the known incidence matrix; and **Z** represents the incidence matrix of random direct additive genetic effect and **e** represents the residual effect associated with each observation. The assumptions for the residual variance were $e\sim N(0,I\sigma_{e}^{2}\;)$ where **I** represent the identity matrix and $\sigma_{e}^{2}2$ the random residual variance. The assumption for the additive genetic effect for the variance components estimation was $a\sim N(0,A\sigma_{a}^{2})$ where $\sigma_{a}^{2}$ is AGV. Once variance components were calculated, genomic information was integrated with pedigree and phenotypes under a single-step GBLUP approach, as described by [8]. In this method, the assumptions for the vector of direct additive genetic effects are distributed as $a\sim N(0,H\sigma_{a}^{2})$. Here, **H** is the unified relationship matrix, which simultaneously incorporates the pedigree relationships of both genotyped and non-genotyped animals [16]. In this approach, a modification of the traditional BLUP is required, replacing the pedigree relationship matrix A with the unified matrix H in the mixed model equations [8].

(2) $$H^{-1}=A^{-1}+\left[\begin{array}{cc} 0 & 0\\ 0 & G^{-1}-A_{22}^{-1} \end{array}\right]$$

where **A^−1^** is the inverse of the additive pedigree-based relationship matrix; $A_{22}^{-1}$ refers to the portion of **A^−1^** that includes only the genotyped animals; G^−1^ is the inverse of the genomic relationship matrix. As **G** is a singular matrix, **G^−^**^1^ was calculated as $0.95\;G+0.05A_{22}^{-1}$ [16].

The genomic relationship matrix was constructed according to the method proposed by [6]

(3) $$G=\frac{MM^{\prime }}{2\sum_{i=1}^{m}p_{i}q_{i}}$$

where **M** is the centered SNP content matrix, $p_{i}$ and $q_{i}$ are the allele frequencies, and mmm is the total number of SNPs. This approach assumes that all SNP effects (u) follow a multivariate normal distribution, which may not reflect biological reality, as causal variants tend to have larger effects.

### 2.4. Genomic Association Analysis

The effects of SNPs were derived from genomic estimated breeding values (GEBVs) for each weighting scenario using the following equation:

(4) $$\hat{u}=\frac{1}{\sum_{j=1}^{m}2p_{j}q_{j}}DM^{\prime }G_{\omega }^{-1}â$$

where **û** is the vector of estimated SNP effects, and â is the vector of genomic estimated breeding values (GEBVs). SNP effects were obtained under each weighting strategy described below, and Manhattan plots were constructed separately for each analytical approach. The identification of genome-wide regions was based on the proportion of AGV explained by non-overlapping windows of 20 adjacent SNPs, defined according to their physical order within each chromosome. Considering the density of the GGP Bovine 50K SNP panel used in this study, genomic windows defined by 20 adjacent SNPs correspond approximately to ~1 Mb across the bovine genome. This estimation was obtained based on the physical distance between the first and last SNP within each window using their genomic coordinates. Given that SNPs in the panel are relatively evenly distributed, this window size provides a consistent genomic resolution and allows the capture of linkage disequilibrium blocks potentially harboring causal variants, particularly in Bos indicus populations where LD may extend over relatively long genomic distances [17]. The variance of each window was calculated as the sum of the SNP variances of all markers contained within the window. Genomic windows explaining ≥0.5% of the total AGV were used for subsequent analyses, including cross-method comparison and identification of functional genes. The use of a threshold was adopted in accordance with previous ssGBLUP-based GWAS that grouped adjacent SNPs into fixed-size windows, with windows comprising 20 SNPs considered an appropriate size according to the literature [18,19]. The value of such a threshold varies across studies and depends on a visual inspection of the Manhattan plots [20]. After SNP effects were estimated, three weighting methodologies were implemented. The Non-Linear A approach was evaluated using three different constants (1.125, 1.2, and 1.5), resulting in five analytical approaches. These methodologies are described in detail below.

#### 2.4.1. Unweighted Method

In the UM, the model assumption is that all SNPs follow the same distribution, meaning that no differentiated weights are assigned to the markers in the construction of the genomic relationship matrix (**G**) matrix [6]. In this procedure, SNP effects (**û**) are calculated directly from the GEBVs obtained via ssGBLUP, without modification by the individual variance of each marker. The G used in this approach is constructed as described previously. The SNP variances were calculated as $\sigma_{u,i}^{2}={\hat{u}}_{i}^{2}\;2p_{i}q_{i}$ where p is the minor allele frequency for the SNP i.

#### 2.4.2. Quadratic Weights

In this methodology, denominated QM, SNP weights were calculated based on the SNP effects obtained from the GEBVs using an iterative procedure, as described by [9]. A diagonal weight matrix **D** was iteratively updated to account for heterogeneous SNP variances, increasing the contribution of SNPs with larger effects and reducing that of SNPs with smaller effects. Allele frequencies were considered in the weighting procedure. The analysis was performed over three iterations, which were determined based on preliminary results and on the literature recommendations. Iterating quadratic weights for more than three rounds tends to result in extreme shrinkage [12,13].

To account for heterogeneous SNP effects, a weighting matrix (**D**) was included, where each diagonal element di corresponds to the variance of SNP i:

(5) $$Var\left(s\right)=D=\left[\begin{array}{cccc} d_{1} & 0 & \ldots & 0\\ 0 & d_{2} & \ldots & 0\\ \vdots & \vdots & \ddots & \vdots \\ 0 & 0 & \ldots & d_{m} \end{array}\right]$$

Based on this, a weighted relationship matrix (G_w_) is defined as:

(6) $$G_{w}=\sum_{j=1}^{m}2p_{j}q_{j}\;MDM^{\prime }\;$$

To account for heterogeneous SNP effects, a diagonal weighting matrix **D** was introduced, where each element $d_{i}$ corresponds to the variance of SNP i. Once genomic predictions are obtained using GBLUP or ssGBLUP, SNP effects can be calculated through a back-solving procedure [6,9,21]:

(7) $$d_{i=}\sigma_{u,i}^{2}\frac{\sum_{j=1\;d}^{m}2p_{j}q_{j}}{\sigma_{a}^{2}}$$

#### 2.4.3. Non-Linear A

The Non-Linear A methodology was proposed by [6] as a computationally efficient approximation of the BayesA method. In this approach, SNP-specific variances are iteratively updated according to:

(8) $$\sigma_{u,i}^{2}=\frac{\sigma_{a}^{2}}{\sum_{j=1}^{m}2p_{j}q_{j}}k{\left(\frac{\left|\hat{u_{l}}\right|}{sd\left(\hat{u}\right)}\right)}^{-2}$$

where $\hat{u_{l}}$ is the estimated effect of SNP i, $\mathrm{sd}\left(\hat{u}\right)$ is the standard deviation of the vector of SNP effects, $p_{i}$ and $q_{i}$ are allele frequencies, $\sigma_{a}^{2}$ is the AGV, and k is a constant controlling the deviation from the normal distribution (k = 1 corresponds to a normal distribution of SNP effects). In the present study, three values of k were evaluated: 1.125 as proposed by ref. [6], 1.2 as suggested by ref. [22] and 1.5. For clarity throughout the manuscript, these approaches are hereafter referred to as A_1.125, A_1.2, and A_1.5, respectively. No upper limit was imposed on SNP weights. All analyses were conducted iteratively over 10 iterations, updating both genomic estimated breeding values (GEBVs) and SNP weights at each step. Convergence was assumed when changes in SNP effects between successive iterations were lower than 0.0001.

### 2.5. Enrichment Analysis

Genomic windows explaining ≥0.5% of the AGV were initially identified for each GWAS methodology considering all 30 chromosomes of the bovine genome (29 autosomes and the X chromosome). The threshold was based on a visual inspection of the Manhattan plots of the unweighted analysis. While arbitrary, such visual inspection can successfully distinguish between noise and a genomic signal. A hypothesis testing using frequentist *p*-values in ssGBLUP exists (Aguilar et al. 2019) [23]. However, the use of *p*-values may not identify differences between regions regarding their substitution effect and may result in false negatives in smaller sample sizes, such as the current study. To avoid false positives due to artifacts, only windows consistently detected in at least two analytical approaches were considered candidate Quantitative Trait Loci (QTL regions and prioritized for gene annotation and subsequent interpretation. Although several regions exceeded the 0.5% threshold within individual approaches, not all were replicated across approaches; therefore, only overlapping windows were retained for QTL interpretation. For a more comprehensive genetic investigation, these recurrent regions were extended by 500 kb upstream and downstream, and genes located within these intervals were identified using the R package (GALLO) [24], by integrating genomic coordinates with gene annotation data obtained from the Ensembl database (Bos taurus reference genome ARS-UCD2.0) using a GTF file. Functional enrichment analysis, however, was performed using genes derived from all genomic windows explaining ≥0.5% of the AGV, regardless of recurrence across methodologies. This broader gene set was used to provide a comprehensive functional overview of the genomic signals detected. The GALLO package was used for enrichment based on Medical Subject Headings (MeSH) terms. Rather than adopting statistical significance thresholds exclusively, interpretation prioritized biological coherence with the studied traits and recurrence of terms across the five analytical approaches.

The same gene list was further explored using the KEGG database through the clusterProfiler package [25]. At this stage, the analysis was performed using ENTREZID codes, with groups defined as conditions. The functions enrichKEGG, enrichGO, and compareCluster were used to explore differences among conditions and to compare functional enrichment for Gene Ontology (GO) categories. Interpretation of pathways and functional categories emphasized biological relevance to carcass traits and recurrence across methodologies rather than relying solely on statistical significance. Graphs were generated using the enrichplot package [25]. Additional packages, including tidyverse and dplyr, were used for data organization [26,27]. This approach provided insights into biological processes, cellular components and molecular functions.

## 3. Results

### 3.1. Description of Phenotypic Data and Heritability

The descriptive statistics and heritabilities of the analyzed phenotypic data are presented in Table 1. The heritabilities (h^2^) ± SD indicate moderate values for both traits studied (0.26 ± 0.05 for REA and 0.22 ± 0.04 for SFT).

### 3.2. Genome Wide Association Study and Identification of Candidate Genes

The genomic windows of 20 adjacent SNPs detected by each GWAS approach for REA and SFT are presented in Supplementary Tables S1 and S2, respectively. For the REA trait, genomic windows explaining ≥0.5% of the AGV were identified for each approach. Using the UM, 10 windows met this threshold, while the QM detected 24 windows. The Non-linear A method identified 13, 16, and 31 windows for weights of 1.125, 1.2, and 1.5, respectively. These results indicate that GWAS approach incorporating SNP weighting, particularly the Non-linear A with a weight of 1.5, were more sensitive in detecting genomic regions associated with additive variance for REA, capturing a greater proportion of genetic variance compared to the UM.

Similarly, for SFT, the UM identified 8 genomic windows exceeding the 0.5% AGV threshold, whereas QM detected 30 windows. The Non-linear A approach identified 11, 14, and 23 windows for weights of 1.125, 1.2, and 1.5, respectively. As observed for REA, SNP-weighted methods increased the number of detected genomic windows, with A_1.5 showing the highest sensitivity in capturing regions explaining a larger proportion of additive genetic variance.

**Figure 1:**
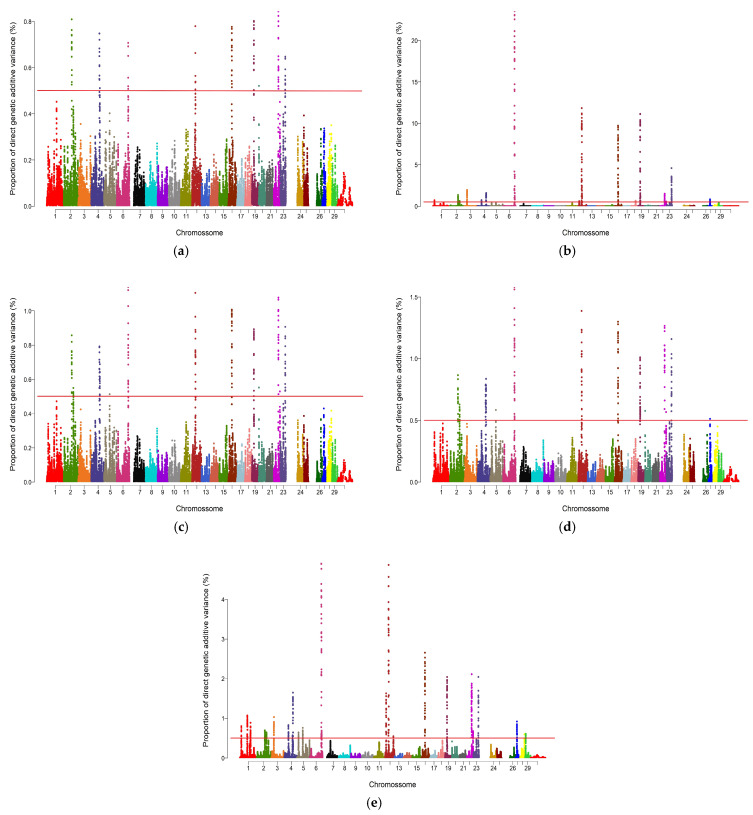
Manhattan plots showing the posterior means of the percentage of AGV explained by genomic windows of 20 adjacent SNPs across chromosomes for the trait Ribeye Area (REA). The five plots correspond to the GWAS approaches: (**a**) UM, (**b**) QM, (**c**) A_1.125, (**d**) A_1.2, and (**e**) A_1.5. Red lines indicate the threshold for significant SNP windows, representing regions explaining at least 0.5% of the AGV. Colors represent different chromosomes. The red vertical line represents the 0.5 % variance explained threshold.

For REA, the UM identified significant regions (≥0.5% AGV) on chromosomes 2, 4, 6, 12, 16, 19, 20, 22, and 23. The QM approach detected regions on chromosomes 1, 2, 3, 4, 6, 12, 16, 18, 19, 22, 23, and 27. The A_1.125 and A_1.2 approaches highlighted largely overlapping sets of chromosomes, including 2, 4, 5, 6, 12, 16, 19, 20, and 22, with A_1.2 additionally detecting chromosome 27. The A_1.5 approach identified the broadest distribution of significant regions, spanning chromosomes 1, 2, 3, 4, 5, 6, 12, 16, 19, 22, 23, 27, 28, and 29. Notably, chromosomes 28 and 29 were exclusively detected by A_1.5. Across all approaches, seven chromosomes (2, 4, 6, 12, 16, 19, and 22) were consistently identified (Figure 1).

**Figure 2:**
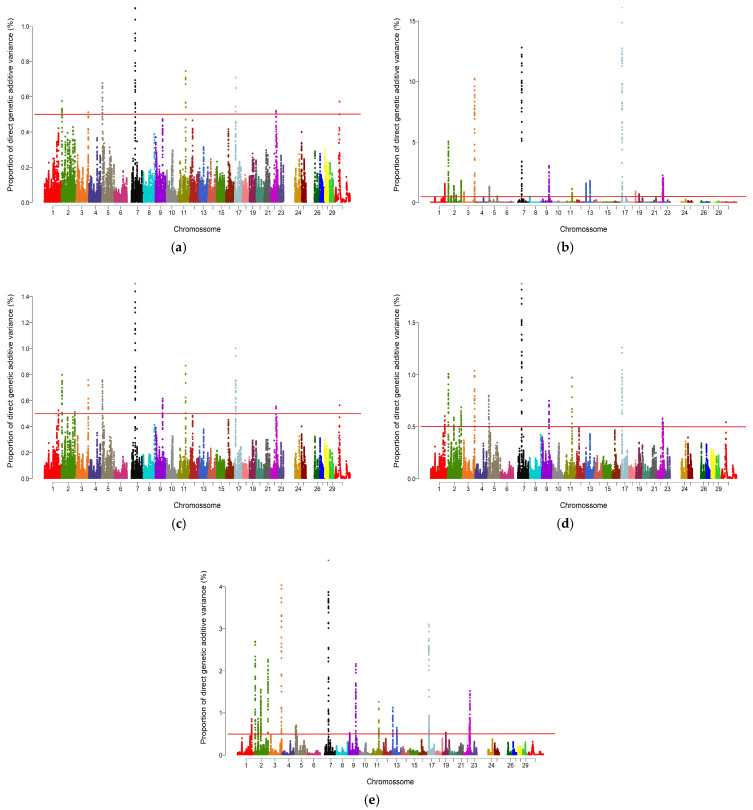
Manhattan plots showing the posterior means of the percentage of AGV explained by genomic windows of 20 adjacent SNPs across chromosomes for the trait Subcutaneous Fat Thickness (SFT). The five plots correspond to the GWAS approaches: (**a**) UM, (**b**) QM, (**c**) A_1.125, (**d**) A_1.2, and (**e**) A_1.5. Red lines indicate the threshold for significant SNP windows, representing regions explaining at least 0.5% of the AGV. Colors represent different chromosomes. The red vertical line represents the 0.5 % variance explained threshold.

For SFT, the UM detected significant regions (≥0.5% AGV) on chromosomes 2, 3, 5, 7, 11, 17, 22, and 30. The QM approach expanded this distribution to chromosomes 1, 2, 3, 5, 7, 9, 11, 13, 17, 18, 19, and 22. The A_1.125 and A_1.2 approaches identified similar sets of chromosomes, including 1, 2, 3, 5, 7, 9, 11, 17, 22, and 30. The A_1.5 approach detected regions on chromosomes 1, 2, 3, 5, 7, 9, 11, 13, 17, 19, and 22. Only QM identified a region on chromosome 18. Seven chromosomes (2, 3, 5, 7, 11, 17, and 22) were shared across all approaches (Figure 2).

**Figure 3:**
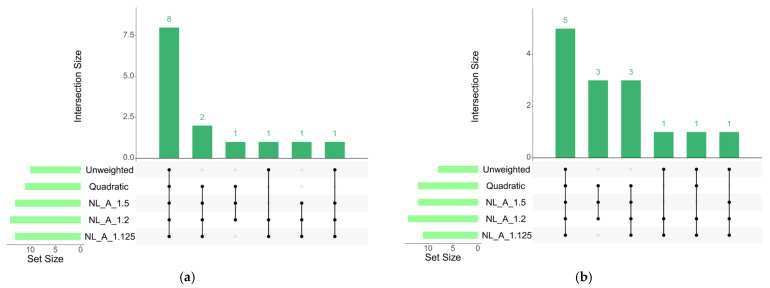
UpSet plots showing the overlapping windows identified in common by the five GWAS approaches (UM, QM, A_1.125, A_1.2, and A_1.5) for (**a**) REA and (**b**) SFT. The horizontal bars on the left represent the total number of windows detected by each approach. The dots and connecting lines below indicate specific combinations of approaches. The vertical bars and the number abovethem show the number of overlapping windows.

The number and distribution of genomic windows explaining more than 0.5% of the AGV (GVA) varied according to the GWAS approach applied. The QM and A_1.5 approaches identified a greater number of windows for both traits analyzed in this study (Figure 1 and Figure 2). These regions were used to identify candidate genes related to each trait, which are listed in Supplementary Table S3 for REA and SFT, respectively. Figure 3 quantifies the windows overlapping across the five GWAS approaches. For REA, 8 windows were detected by all methods, while for SFT5, windows overlapped across all methods. Only windows identified by at least three methods were considered stable.

### 3.3. Functional Analysis and Pathway Enrichment

The KEGG network enrichment analyses were performed for two traits considering the five GWAS approaches. For REA, 165 pathways were identified using the UM, 203 with QM, 197 with A_1.125, 203 with A_1.2, and 239 with A_1.5 (Supplementary Table S4). Among the most relevant pathways for REA were the mTOR signaling pathway (bta04150), detected across all GWAS approaches, and the Growth hormone synthesis, secretion and action pathway (bta04935), also identified in all analyses. Both are modulated by the *AKT3* gene, consistently detected across all approaches. These pathways are related to the regulation of muscle growth, energy metabolism, and cell proliferation, reinforcing the functional importance of these mechanisms and the associated genes for the REA trait.

For SFT, several pathways were identified, among which some stood out due to their relevance and recurrence across multiple GWAS approaches. Using the UM, 30 pathways were identified; with QM, 207 pathways; with A_1.125, 60 pathways; with A_1.2, 98 pathways; and finally, with A_1.5, 128 pathways (Supplementary Table S5). Among the pathways directly related to the trait, the PPAR signaling pathway (bta03320), which directly regulates adipogenesis, stood out and was present in all GWAS approaches except A_1.5, with *ACOX2* identified as the enriched gene for this pathway across the approaches in which it appeared. Another relevant pathway is Insulin resistance (bta04931), detected in all GWAS approaches, with *GFPT1* identified as the enriched gene for this pathway.

The functional enrichment analysis of MeSH terms for REA revealed 109 terms with the UM, 275 with QM, 325 with A_1.125, 351 with A_1.2, and 305 with A_1.5 (Supplementary Table S6). Some terms stood out for being detected by multiple GWAS approaches and for directly influencing REA, including Amino Acids (D000596), identified by three approaches (UM, A_1.125, and A_1.2). This term is fundamental for REA, as amino acids are the structural units of proteins and, therefore, essential for muscle growth. Another highlighted term is IGF (Insulin-like Growth Factor) (D007334), detected by all GWAS approaches, indicating a consistent association with the trait, regardless of the approach used. This hormone plays a fundamental role in muscle growth and differentiation (Supplementary Table S12).

For SFT, the analysis revealed 61 MeSH terms with the UM, 262 with QM, 115 with A_1.125, 156 with A_1.2, and 200 MeSH terms with A_1.5 (Supplementary Table S7). Among the terms detected by multiple methods and with a direct effect on the trait, are Follistatin (D038681) and Carnitine (D002331), both identified by 3 GWAS approaches, the UM, A_1.125, and A_1.2, indicating a strong genetic association with SFT. These terms play fundamental roles in lipid metabolism and, consequently, in subcutaneous fat deposition (Supplementary Table S13).

**Figure 4:**
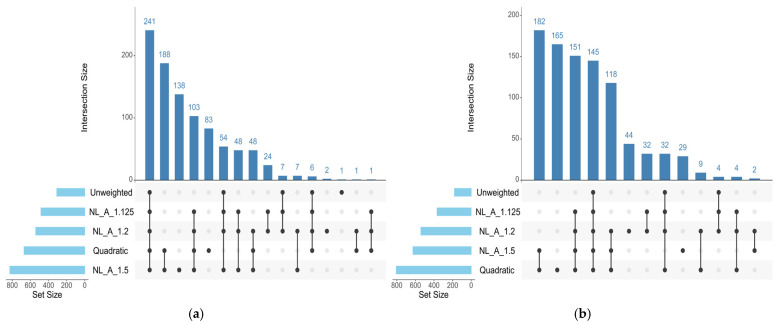
UpSet plots showing the intersection of enriched GO:BP terms identified by the five GWAS approaches (UM, QM, A_1.125, A_1.2, and A_1.5) for (**a**) REA and (**b**) SFT. The horizontal bars on the left represent the total number of GO:BP terms detected by each approach. The dots and connecting lines below indicate specific combinations of approaches. The vertical bars and numbers above them show the number of shared GO:BP terms.

Another enrichment analysis performed in our study was based on Gene Ontology for Biological Processes (GO:BP), which allowed the identification of relevant terms for both traits analyzed (Figure 4). For the trait REA, 309 terms were identified with the UM, 671 with QM, 484 with the non-linear weighted method A_1.125, 542 with A_1.2, and 827 with A_1.5 (Supplementary Table S8). Among these terms, some stand out due to their direct impact on REA and because they were detected by more than three GWAS approach (Supplementary Table S5). First, the GO:BP term actin cytoskeleton organization (GO:0030036) is highlighted, as it is essential for muscle growth. This term was present in all GWAS approaches evaluated, demonstrating its strong association with the trait. Another relevant term is actin filament bundle organization (GO:0061572), identified in UM and all non-linear approaches. This term is directly associated with the structuring of muscle cells and, consequently, with REA.

For SFT, 181 GO:BP pathways were identified with the UM, 806 with QM, 368 with A_1.125, 541 with A_1.2, and 627 with A_1.5. Weighted GWAS approaches, particularly QM and A_1.5, detected more biologically relevant pathways than the less-weighted or UM (Supplementary Table S9). Among the identified GO:BPs, lipid homeostasis (GO:0055088) stands out for its role in regulating lipid synthesis and degradation, directly impacting SFT. This term was detected by all GWAS approaches evaluated, highlighting its functional relevance for the trait regardless of the method used. Another notable term is regulation of lipid metabolic process (GO:0019216), which is important for SFT as it influences lipogenesis and fat storage, and was also identified by all GWAS approaches, reinforcing its functional relevance in the regulation of the phenotype.

**Figure 5:**
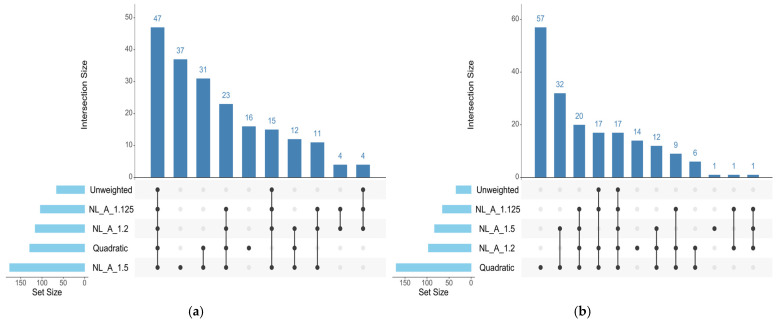
UpSet plots showing the intersection of enriched GO: CC terms identified by the five GWAS approaches (UM, QM, A_1.125, A_1.2, and A_1.5) for (**a**) REA and (**b**) Subcutaneous SFT. The horizontal bars on the left represent the total number of GO:CC terms detected by each approach. The dots and connecting lines below indicate specific combinations of approaches. The vertical bars and the number above them show the number of shared GO:CC terms.

Additionally, a Gene Ontology Cellular Component (GO:CC) enrichment analysis was performed, which identified terms relevant to both analyzed traits. Some terms stood out for their direct influence on the studied trait and for being detected simultaneously by multiple GWAS approaches (Figure 5). For REA, 66, 129, 104, 116, and 176 GO:CC terms were detected with the UM, QM, A_1.125, A_1.2, and A_1.5 approaches, respectively (Supplementary Table S10). Among the highlighted terms, stress fiber (GO:0001725) and actomyosin (GO:0042641) were prominent, as they are directly involved in the regulation of muscle hypertrophy and the organization of contractile structures. These components were detected by all GWAS approaches evaluated, demonstrating their consistency and strong association with REA.

Regarding SFT, 34 significant terms were detected with the UM, 170 with QM, 65 with A_1.125, 97 with A_1.2, and 83 with A_1.5 (Supplementary Table S11). Among all GO:CC terms enriched for SFT, peroxisome (GO:0005777) and endosome (GO:0005768) stand out, both directly related to the phenotype due to their fundamental role in fatty acid and lipid metabolism. These GO:CCs were detected by all GWAS approaches evaluated, except A_1.5, highlighting their functional relevance in the regulation of the trait.

**Figure 6:**
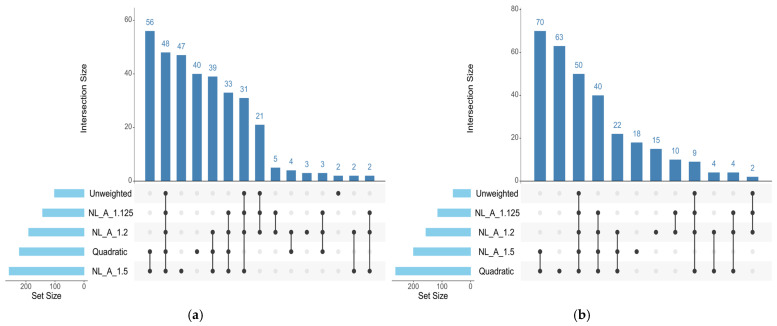
UpSet plots showing the intersection of enriched GO: MF terms identified by the five GWAS approaches (UM, QM, A_1.125, A_1.2, and A_1.5) for (**a**) REA and (**b**) SFT. The horizontal bars on the left represent the total number of GO: MF terms detected by each approach. The dots and connecting lines below indicate specific combinations of approaches. The vertical bars above show the number of shared GO: MF terms.

The latest enrichment analysis was performed using Gene Ontology Molecular Function (GO:MF), which identified relevant terms for both studied traits (Figure 6). For REA, 102 terms were detected with the UM, 223 with QM, 143 with A_1.125, 191 with A_1.2, and 258 with A_1.5 (Supplementary Table S12). Among the identified terms, some stand out for directly influencing the studied phenotype and for being detected across multiple GWAS approaches, highlighting their consistency. Notably, calcium/calmodulin-dependent protein kinase activity (GO:0004683), fundamental for muscle growth, was identified in all methods except QM, and actin binding (GO:0003779), which plays a crucial role in the structure and stability of muscle fibers.

Regarding the trait SFT, the analysis identified 61 GO:MF terms with UM, 262 with QM, 115 with A_1.125, 156 with A_1.2, and 200 with A_1.5 (Supplementary Table S13). Among the most relevant GO:MFs, lipid binding (GO:0008289) stands out, as it directly influences subcutaneous fat accumulation and was detected in all evaluated approaches. Another important term is growth factor activity (GO:0008083), which stimulates adipogenesis and was identified in four of the five approaches, reinforcing its association with the SFT phenotype. In both cases, the QM and A_1.5 approaches were the most efficient in detecting GO:MF terms.

## 4. Discussion

### 4.1. Phenotypic Data and Heritability

The estimated heritability for REA (0.26) (Table 1) was classified as moderate. Similar values were reported (0.29) in Guzerá cattle [28,29]. In other Zebu breeds, heritability estimates have been reported to range from 0.24 to 0.46, including Brahman [30], Tabapuã [31], and Nelore [32]. Thus, the estimate obtained here is consistent with previous studies, falls within the Zebu range, and supports the feasibility of direct genetic selection for REA and the identification of genomic regions via GWAS.

For SFT, heritability was moderate (0.22) (Table 1). Similar studies in Guzerá cattle reported lower estimates (0.10) [28,29]. The higher value observed likely reflects greater additive genetic variability or improved control of environmental effects. In Nelore cattle, heritability estimates reported fall within one standard error of the present estimate [33,34,35], indicating consistency within the breed group and supporting GWAS-based identification of genomic regions and their application in genetic selection. These estimates were obtained primarily to support the GWAS analyses through the estimation of additive genetic variance.

### 4.2. Different Weightings and the Identification of Genomic Windows

The results obtained within each of the weighting approaches followed the expected behavior described in the literature. The unweighted methods resulted in a ‘flat’ Manhattan plot, suggesting a polygenic scenario. Differently, the quadratic weights resulted in few windows explaining a large proportion of variance. Finally, the Non-linear A approach resulted in intermediate graphs, depending on the equation parameters. Oligogenic traits tend to achieve better results between 3 and 5 iterations of quadratic weights [12,13,36]. In very simple genetic architectures, windows explaining up to 60% of the genetic variance resulted in higher accuracies across weighting approaches [12]. However, for more polygenic traits, the extreme shrinkage harms prediction and discovery, and un-weighted or Non-linear A approaches with more conservative parameters tend to converge at higher accuracies [37]. Because the causative variants are unknown and GWAS peaks may fluctuate across methods [13] and subsamples of population [18], using the genomic windows in common across multiple approaches is expected to reduce the incidence of artifacts. In the current dataset, subsetting is not feasible due to the reduced sample size. Therefore, the multiple approaches for GWAS should control the incidence of artifacts.

In both traits, the UM identified the fewest significant windows, with only 10 genomic windows explaining more than 0.5% of the GVA in REA and only 8 in SFT, whereas the approaches that incorporate weights into SNP effects showed greater sensitivity, detecting a significantly higher number of windows capable of explaining relevant proportions of GVA for the phenotypes, especially QM and A_1.5 (Supplementary Tables S1 and S2). This occurs because non-weighted methods assume the same prior variance for all SNPs, that is, they treat each marker as equally likely to have an effect [13]. This makes the approach more conservative, detecting only the strongest and most evident signals.

The assumption that all SNPs contribute equally to the expression of a phenotype may be biologically incorrect, because some SNPs are located in regions with genes directly involved in muscle development and fat deposition, including causal variants, while others are located in neutral regions or regions with little functional impact [38,39]. When non-weighted markers are used to identify associations, genetic heterogeneity is disregarded, diluting relevant effects among thousands of other markers with null or near-zero effects [40]. Furthermore, the highly polygenic nature of REA and SFT implies that many loci with small to moderate effects, including causal variants, are unlikely to be captured by UM.

In the present study, QM stood out by identifying the highest number of significant windows for SFT and the second highest for REA, behind only A_1.5. The results of this study demonstrate that the greater number of windows detected by the weighted methods, for both REA and SFT, is directly associated with the approaches SNP weighting schemes. In QM, the weight assigned to each marker is proportional to its own estimated effect and its allelic variability [9]. In this way, SNPs that initially show association with the phenotype have their signal reinforced throughout the iterations, whereas SNPs with effects close to zero lose influence, which favors greater detection of significant windows. However, ref. [10] observed that although QM may initially increase the signal of some SNPs, it tends to exhibit instability in weight updates, leading to extreme changes between iterations. Such behavior can increase random fluctuations in the estimated effects, leading to inflation of false positives and reducing the reliability of detecting relevant regions.

Regarding the weighted Non-linear A approaches, across both traits analyzed, our study revealed distinct responses for each of the three evaluated approaches, with a gradual pattern across the weighting levels. When applying A_1.125 in our study, initially proposed by [6]. A modest increase in the number of detected windows was observed relative to UM for both REA and SFT, although it remained lower than that identified by QM and the other weighted methods. This reflects the moderate nature of this adjustment and its greater stability throughout the iterations used [41,42].

However, a study conducted by [43] on morpho-functional traits in dairy cattle highlighted that the differences between GEBV predictions and the reliabilities estimated by the non-weighted approaches and A_1.125 were insignificant, indicating that both approaches provided equally reliable estimates. A study by [44], showed similar results in terms of accuracy when using the constant 1.125 for SNP weighting; the authors did not observe improvement in the prediction of the studied traits, obtaining accuracy values similar to those of the non-weighted approach. This compromises the detection of genomic windows, since the lack of gain in GEBV accuracy limits the differentiation of SNP effects, reducing the ability to identify genomic regions significantly associated with the traits.

Given this, increasing the weighting constant could produce beneficial effects. In the present study, when A_1.2 was applied, as proposed by [22], a slight increase in the number of detected genomic windows was observed for both REA and SFT compared to the UM, and a slightly greater increase than with A_1.125. These results are consistent with those reported by [45] who demonstrated that, for most carcass and meat traits in Hanwoo cattle, Non-linear A approaches with CT 1.125 and 1.25 resulted in nearly identical predictions, with only a small increase observed in specific iterations and phenotypes.

Similarly, ref. [12] observed that a moderate increase in CT in the Non-linear A method tends to improve the distinction among SNPs with larger effects; however, this gain becomes more evident when the trait presents loci with moderate effects. For highly polygenic traits, the impact of weighting tends to be smaller, which aligns with the observed results. Although several studies in beef cattle commonly use CT values between 1.125 and 1.25 for Non-linear A approaches [45,46,47], higher values close to 1.5 have been explored in Holstein dairy cattle [10]. Increasing CT may further enhance genomic signals, potentially raising the number of detected windows and improving the identification of relevant regions. However, excessive weighting can also generate extreme estimates and shrink smaller effects, increasing bias and the risk of false-negative associations.

In the present study, A_1.5 was the approach that identified the greatest number of significant genomic windows for both traits, surpassing all other approaches. These results indicate that increasing the weighting constant intensifies the differentiation among SNPs, enhancing the contrast between those with more relevant effects and reducing the influence of markers with very small contributions [6,22]. As a result, A_1.5 favors the identification of genomic windows with greater capacity to explain additive genetic variability, particularly in traits influenced by many loci with small to moderate effects, such as REA and SFT [48,49]. However, the increase in sensitivity observed with more intensive weighting approaches, such as A_1.5 and QM, may compromise the statistical stability of the estimates.

Differences in GWAS peak locations across approaches may be due to multicollinearity among markers. This occurs because SNPs that are in high linkage disequilibrium (LD) tend to present high correlation with each other and start to carry redundant genetic information, making it difficult to separate the individual contribution of each marker statistically [50,51]. Thus, the variance inflation factor is higher when LD is stronger resulting in potential spurious associations [52]. This intensity effect is evident in QM, because the iterative process and the extreme weights caused by the way the maker variance is calculated can amplify the effects of the dependency relationships among correlated markers [10].

Moreover, cattle populations frequently exhibit long-range linkage disequilibrium (LD), reflecting reduced effective population sizes (Ne), as reported by [53,54]. This pattern may be related to the intensive use of a limited number of superior sires, strong directional selection, and demographic histories characterized by a reduced number of founders in zebu breeding populations [54]. Although specific estimates of effective population size (Ne) for Guzerá cattle are limited, studies in other zebu breeds suggest that increases in census population size have not necessarily been accompanied by maintenance of genetic diversity [55]. Therefore, it is plausible that similar demographic dynamics could contribute to reduced Ne and the formation of extensive LD blocks in Guzerá populations, resulting in redundancy of genomic information among neighboring SNPs. According to [11] a moderate Ne limits the ability of models to differentiate the effects of highly correlated markers. This effect is consistent with the findings of ref. [56], who observed that an increase in the number of detected regions does not guarantee greater precision in QTL localization, especially when extensive LD blocks cause neighboring SNPs to carry redundant information.

Thus, approaches that involve iterative reweighting, particularly those applying higher weights, may amplify signals in regions with redundant information, leading to less stable and potentially inflated estimates. Evidence of inflation bias associated with weighting strategies informed by GWAS results has been reported by [57] suggesting that intensive weighting may compromise estimate stability compared to unweighted approaches. Consequently, artificially expanded windows and reduced ability to distinguish adjacent regions with different effects may occur, compromising precision in identifying truly associated genomic regions. Additionally, windows may shrink toward zero due to the exponential nature of the weights, potentially increasing the number of false-negative windows.

Reductions in prediction reliability under weighted models have also been reported in cattle populations, where quadratic weighting reduced reliabilities [10] and weighted ssGBLUP models showed slightly lower reliability compared to basic ssGBLUP models [58], reinforcing the potential instability associated with intensive weighting. Although weighting increases detection sensitivity, it may be accompanied by greater statistical instability, particularly in populations with strong LD and moderate to low effective population size. Therefore, no method can be considered entirely superior. Each approach presents a distinct balance between sensitivity and stability. Thus, the choice of the most appropriate method depends on both the genetic architecture of the trait and the structure of the analyzed population.

### 4.3. Recurrent Genomic Regions and Candidate Genes Across Approaches

The Manhattan plots for REA (Figure 1) illustrate the contrast between the approaches regarding their sensitivity in detecting associated regions. The UM (a) presents few peaks above the 0.5% of AGV threshold, highlighting its conservative nature. In contrast, the weighted methods, particularly QM and A_1.5, exhibit a greater number and intensity of distinct peaks, resulting from the iterative process that enhances the signals of the most relevant SNPs. However, peak broadening is observed in chromosomes possibly under strong linkage disequilibrium, such as BTA6 and BTA12, indicating potential signal redundancy and lower precision in delimiting causal regions. BTA6, however, maintained a high peak across all approaches, indicating a robust genetic signal and the presence of variants with consistent effects on REA. This pattern is consistent with the findings of [17], who reported lower allelic variability and high LD values in BTA6, as well as long average distances between SNPs in BTA12, in Nelore and non-Zebu populations, respectively. Such behavior reinforces the previously discussed relationship between sensitivity and precision in the identification of genomic windows. Conditioning GWAS hits to genotypes of the peak SNP may help identify if there is a single or multiple QTL signal and to generate a confidence interval to the QTL [59].

Regarding SFT (Figure 2), the Manhattan plots show a pattern similar to that observed for REA, highlighting differences among the approaches in their sensitivity to detect relevant windows. The UM shows few peaks above the threshold, again reflecting its conservative nature. The weighted methods, especially QM and A_1.5, show a higher number and intensity of peaks across the genome, a result also attributed to the iterative process, which reinforces the signals of markers with higher estimated effects. Peak elongation is observed in specific regions, particularly BTA3, BTA7, and BTA17, suggesting possible signal redundancy and, consequently, lower precision in identifying relevant windows. BTA7 stands out, maintaining a consistent peak across all approaches, suggesting a stable genetic signal and the presence of variants with real effects on SFT. The involvement of BTA7 in phenotypes related to fat deposition was thoroughly reported by [60], in studies with Wagyu cattle, while ref. [17], observed a high proportion of rare alleles in BTA7 and BTA17 in Nelore animals, indicating regions potentially under strong LD. Thus, the SFT plots corroborate the relationship between sensitivity and precision in detecting important genomic regions, similar to that observed for REA.

Although the Manhattan plots demonstrate the specific behavior of each approach with respect to sensitivity and accuracy in detecting relevant regions, the joint comparison of results allowed more accurate identification of recurrent regions and genes across approaches (Supplementary Table S3).

For REA, some genes were recurrently identified across all GWAS approaches, mainly in the regions BTA6, BTA16, and BTA19 (Supplementary Table S3). Among them, *AKT3* (AKT serine/threonine kinase 3) stood out, suggesting a strong association with the analyzed trait. This gene responds to growth factor stimuli such as insulin and IGF, promoting an increase in cell size by stimulating protein synthesis and inhibiting degradation the PI3K/AKT/mTOR pathway. This pathway participates in growth factor signaling, muscle hypertrophy, and lipid metabolism, highlighting *AKT3* as a crucial regulator of muscle development in Angus beef cattle [61]. Another gene identified in all approaches was *NOS2* (nitric oxide synthase 2). Although frequently studied in the context of immunity [62,63], its role in nitric oxide production, which regulates blood flow and cellular metabolism, provides a functional basis for its association with REA.

In addition, *CLNK* and *ZNF518B* were also recurrently detected, but their functions in cattle are not yet well characterized. *CLNK* is associated with immune signaling in other species [64], whereas *ZNF518B* is described as a regulator of gene expression in humans [65]. These findings suggest only a potential genomic relevance, which requires further investigation in future studies in beef cattle.

For SFT, the integrated analysis of the GWAS approaches revealed a set of genes recurrently identified across all approaches, located mainly in the BTA2, BTA7, and BTA11 regions (Supplementary Table S3). Among them, *MSTN* (myostatin) stands out, having been consistently detected by the three most conservative approaches (UM, A_1.125, and A_1.2). Because these approaches tend to identify more robust signals either by not weighting SNP effects, as in the case of UM, or by applying milder iterative reinforcement of marker effects, as in A_1.125 and A_1.2 the presence of *MSTN* among them suggests that this region carries a relevant and biologically meaningful effect on SFT. This gene is widely recognized as a negative regulator of muscle growth. In cattle, a study involving Korean breeds reported that the promoter polymorphism g.-371T>A in *MSTN* was significantly associated with meat quality and subcutaneous fat thickness [66]. Similarly, a study in pigs demonstrated that functional reduction in *MSTN* also alters lipid metabolism, increasing subcutaneous fat accumulation [67]. These findings support the functional relevance of *MSTN* for the SFT phenotype.

In addition to *MSTN*, the genes *TNFAIP8* and *DMXL1* also stood out. Although these genes were identified as recurrent across all GWAS approaches for SFT (Supplementary Table S3), no published studies linking them directly to subcutaneous fat deposition in cattle were found. However, studies in other mammals indicate that *TNFAIP8* can regulate inflammatory pathways and lipid metabolism. A study conducted in mice demonstrated the role of this gene in modulating enzymes involved in fatty acid metabolism [68]. Furthermore, work by [69], showed that *TNFAIP8* plays a sex-dimorphic role in adipose tissue, influencing *PPARG* and the distribution of fat depots. Additionally, studies in mammals have shown that *DMXL1* participates in lysosomal homeostasis and V-ATPase assembly, cellular processes associated with energy metabolism, and potentially with subcutaneous fat deposition [70,71].

The recurrence of these genes across the different GWAS approaches reinforces the hypothesis that they may be relevant to the genetic variability of SFT. Their repeated identification across distinct statistical approaches also supports the consistency of the detected signals, indicating that they warrant functional investigation in future studies and potential consideration in genomic selection strategies for subcutaneous fat deposition in beef cattle.

### 4.4. Functional Enrichment Analysis of Genomic Regions Associated with REA and SFT

Functional enrichment analyses were conducted to explore functional categories and biological pathways associated with genes located within genomic regions detected by the different GWAS approaches. Considering that one of the objectives of this study was to compare the biological signals identified by different GWAS methodologies, the interpretation of enrichment results was based on the biological relevance of functional terms to the traits evaluated and on the recurrence of these terms across the five analytical approaches. Thus, unlike traditional enrichment strategies that prioritize terms based on *p*-values, in this study the terms were selected and discussed according to their biological plausibility and consistency across the different GWAS methodologies [72]. This strategy allowed the identification of functional categories and biological pathways consistently associated with genomic regions detected by multiple GWAS models, highlighting potentially robust functional mechanisms underlying carcass traits.

The functional interpretation of genes identified in the GWAS analyses can be further enhanced through enrichment approaches, such as KEGG, which have already been employed in relevant studies with beef cattle [73,74,75]. This type of analysis allows the identification of metabolic pathways potentially involved in the expression of the studied traits and assesses whether different GWAS approaches converge on common biological mechanisms. For both traits, GWAS approaches with more intensive weighting identified the largest number of KEGG pathways. The non-linear weighted approach A_1.5 identified the highest number of pathways for REA, whereas for SFT, the approach most sensitive in detecting pathways was QM, reinforcing a pattern already observed in other analyses in this study. Accordingly, pathways identified in at least three of the five studied approaches and directly related to the phenotypes’ physiology were considered, as they help reinforce the biological robustness of the findings while avoiding interpretations based on sporadic signals or those specific to a single statistical model.

One of the main limitations of GWAS for complex traits in livestock is the instability of the identified windows. Such problems were previously described by [18] and can be observed when comparing genes identified in different studies. The genes located in those regions may provide insight into the biological mechanisms involved in heat tolerance, as well as their GO terms. However, SNP effects and variance may differ by method of choice, as observed in windows that did not overlap across the different weighted approaches used in this study. In rainbow trout, weighted ssGBLUP and BayesB differed in SNP windows in GWAS in the same dataset [76]. Additionally, association studies with RAD SNPs in the same population did not identify any QTL in common [77]. Such changes may be a consequence of the small number of independent chromosome segments (*q*) in selected populations with limited effective population size (*Ne*). The estimated number of *q* is (2×Ne×L/log(2Ne×L) [78,79]. Using the effective population size of 104 [80] do Ne calculated for Guzerá cattle and a chromosome length of 30 M [81], would mean that only 1644 independent chromosome segments exist. With the number of markers around 50k, it would increase the predictor error variance of SNP effects due to multicollinearity. While the limited dimensionality of genomic information can reduce computational power for genomic predictions [82], it may result in changes in GWAS peaks due to variation in SNP effects when different methods or data are used. Another solution for evaluating such instability would be repeated subsampling for GWAS. However, such an approach is not ideal for a small sample size, since it may increase problems related to reduced power. Therefore, only genes identified in more than 3 methods were discussed to avoid including genomic regions that would be included due to spurious associations.

For REA, two pathways identified through KEGG enrichment stand out. The first is the mTOR signaling pathway (bta04150), which is known for regulating muscle hypertrophy by integrating signals for growth, energy, and nutrients. Studies in mammals have shown that activation of the PI3K/AKT cascade, which subsequently activates mTORC1, stimulates protein synthesis through its downstream effectors, such as p70S6K and 4E-BP1, whereas inhibition of mTORC1 by rapamycin prevents an increase in muscle fiber size [83,84]. In cattle, the PI3K/AKT/mTOR pathway also plays an important role in the regulation of myocyte growth and differentiation, being activated by myostatin mutation via RACK1 [85]. These findings demonstrate the fundamental role of this pathway in muscle hypertrophy and, potentially, in REA variation.

Another pathway identified for REA and highly relevant to this phenotype is the Growth Hormone synthesis, secretion, and action pathway (bta04935). This pathway plays an important role in regulating body growth and muscle hypertrophy, linking Growth Hormone (GH) to the expression of Insulin-like Growth Factor 1 (IGF-1), which acts on myocyte differentiation and proliferation. Studies in beef cattle have shown that polymorphisms in the Growth Hormone Receptor (*GHR*) gene are associated with longissimus muscle area, consequently with REA and other relevant carcass traits [86,87]. Experimental studies demonstrate that GH directly stimulates protein synthesis in bovine muscle cells, thereby demonstrating its anabolic activity independent of local IGF-1 [88,89].

Furthermore, a study by [90] associates *GHR* as a candidate gene for traits related to meat production and quality in cattle, reinforcing that the GH/IGF-1 pathway plays a fundamental role in the regulation of muscle growth and, consequently, REA. The gene enriched in both aforementioned pathways was *AKT3*, previously detected as a common gene within an important genomic region identified by all five GWAS approaches. This reinforces the convergence between the genomic regions highlighted by GWAS and the molecular pathways identified through functional enrichment, corroborating the strong association of this gene with REA.

Regarding SFT, a prominent enriched pathway is the PPAR signaling pathway (bta03320). This pathway is relevant to this trait because it is involved in the differentiation of preadipocytes into adipocytes, a fundamental process of adipogenesis [91]. An in vitro study by [92] showed that insulin, together with PPARγ agonists, significantly increased the expression of genes regulating adipogenesis and the accumulation of triglycerides in bovine preadipocytes, highlighting the PPAR pathway as a regulator of adipogenic differentiation. This information suggests that PPAR signaling, when activated in cattle, can promote lipid accumulation, which may contribute to subcutaneous fat deposition. The gene enriched in this pathway was *ACOX2*, which encodes a peroxisomal enzyme involved in fatty acid oxidation, modulating lipid metabolism, and thus influencing subcutaneous fat accumulation. A study by [93] in Wagyu–Angus cattle, similarly, this gene was highlighted as a candidate within the PPAR pathway, suggesting its association with SFT, which corroborates the findings of our study.

Another prominent enriched pathway for SFT was the Insulin resistance pathway (bta04931). This pathway integrates metabolic signals regulating lipolysis and lipid storage in adipose tissue, with insulin acting as an anabolic hormone that stimulates glucose uptake and triglyceride synthesis in adipose tissue while suppressing lipolysis [94,95]. Thus, this pathway can alter adipose tissue sensitivity, increasing systemic lipolysis when resistance is present or promoting local lipid accumulation when it is impaired. A transcriptomic study in bovine adipose tissue, combined with integrated transcriptional and metabolomic analyses, highlighted that alterations in pathways associated with insulin resistance directly influence metabolic processes that modulate fat deposition [96]. Furthermore, another study using the muscle transcriptome of F1 Angus-Nellore cattle also identified the insulin resistance pathway as involved in lipid metabolism associated with fat regulation [97]. These pieces of evidence reinforce the notion that modulation of this pathway plays a relevant role in SFT in cattle.

*GFPT1* was the gene identified for the insulin resistance pathway, detected recurrently across the GWAS approaches. This gene, by encoding glutamine-fructose-6-phosphate amidotransferase, limits the hexosamine biosynthetic pathway (HBP), a metabolic pathway that diverts part of glucose to generate UDP-GlcNAc, a substrate for O-GlcNAc protein modification [98,99]. A study by [100], demonstrated that increased glucose flux directed to the HBP acts as a mechanism inducing insulin resistance, especially in adipocytes, impairing insulin-stimulated glucose uptake and directly affecting the balance between lipogenesis and lipolysis, potentially impacting SFT.

In addition to KEGG pathway enrichment, MeSH term enrichment was also performed. This type of analysis allows the identification of broad and hierarchical biological categories associated with genes located within relevant GWAS regions. Unlike the KEGG approach, MeSH terms group physiological, metabolic, anatomical, and biochemical information derived from the biomedical literature, facilitating the interpretation of how genes relate to processes involved in trait expression [101]. Thus, this tool can complement biological validation, highlight recurring themes across GWAS approaches, and inform inferences drawn from the detected genomic regions.

For REA, the most sensitive approach in detecting MeSH terms was A_1.2, suggesting that an intermediate weighting method can also yield a greater number of relevant terms in enrichment analyses. This is important, as this weighting tends to minimize inflated signals that are not directly associated with the phenotype. The enrichment revealed terms that are important and directly associated with muscle development; moreover, these terms showed high recurrence across GWAS approaches, reinforcing their strong association with physiological processes influencing this trait. One of these terms is Amino Acids (D000596), which is biologically plausible, as amino acids constitute the structural building blocks of muscle proteins and are essential for protein synthesis during skeletal muscle development [102]. A study in cattle demonstrated that amino acids such as arginine and lysine can stimulate muscle hypertrophy through activation of the mTOR pathway, evidencing their anabolic function and importance in skeletal muscle development [103].

Another relevant term detected was Insulin-like Growth Factor (IGF) (D007334). A study conducted with bovine satellite muscle cells demonstrated that IGF-1 overexpression increases the expression of myogenic markers, such as MyoG and MyoHC, through activation of the PI3K/AKT pathway, which corroborates its fundamental role in muscle tissue expansion and development [104]. Thus, both terms highlight essential mechanisms for skeletal muscle development, reinforcing their importance for REA.

Regarding SFT, the approach that identified the most enriched MeSH terms was QM. As an approach with stronger weighting, it tends to be more permissive, capturing a greater number of responses, albeit at the risk of inflated results. The MeSH enrichment analysis identified terms relevant to subcutaneous fat deposition, particularly Follistatin (D038681) and Carnitine (D002331), both detected by three of the five evaluated GWAS approaches (UM, A_1.125, and A_1.2). The detection of Follistatin is consistent, as this glycoprotein acts as a myostatin antagonist, modulating muscle growth and processes related to lipid metabolism [105,106]. A study in mice demonstrated that follistatin overexpression reduces the activity of inhibitory pathways of adipocyte differentiation and alters body fat accumulation [107].

The term Carnitine is also relevant, as carnitine plays a central role in the transport of long-chain fatty acids into the mitochondria, an essential step for β-oxidation and the regulation of lipid accumulation. This process is catalyzed by the enzyme Carnitine Palmitoyltransferase 1B (CPT1B) [108,109]. A study by [110], demonstrated an association between polymorphisms in CPT1B and dorsal fat thickness in Simmental cattle, indicating that the efficiency of the carnitine-dependent pathway can directly influence lipid accumulation. Therefore, these terms represent crucial processes of lipid metabolism involved in subcutaneous fat deposition and consequently affect SFT.

In addition to KEGG and MeSH enrichment analyses, GO based enrichment was also performed, an essential approach that allows structured description of Biological Process, Cellular Component, and Molecular Function associated with genes identified in GWAS [111,112]. Accordingly, only GO terms with a direct and plausible functional relationship with the analyzed phenotypes and detected simultaneously by at least three ssGWAS approaches were considered, as repetition across approaches reinforces the consistency of the findings of this study. For REA, A_1.5 was the approach that identified the most GO terms across all aspects, whereas for SFT, QM was the most efficient method in detecting GO terms in all aspects as well (Figure 4, Figure 5 and Figure 6). This occurs because these approaches enhance the effects of the most informative SNPs, increasing sensitivity to complex associations and consequently increasing the likelihood of identifying associated genes and more functional GO terms.

For REA, numerous GO:BPs were detected recurrently across GWAS approaches, among them actin cytoskeleton organization (GO:0030036) and actin filament bundle organization (GO:0061572). They are also notable for their high relevance to the studied phenotype, as both processes can regulate myofibril assembly and maintenance, as well as the arrangement of muscle fibers. While GO:0030036 refers to the general organization of the actin cytoskeleton, GO:0061572 specifically describes the formation of actin filament bundles, which are crucial for maintaining the structural integrity and shape of muscle fibers, mediated by actin-binding proteins such as α-actinin [113]. According to [114], actin filaments in sarcomeres are highly dynamic, and regulation of their polymerization, depolymerization, and stabilization ensures proper myofibril formation and maintenance of their functional integrity. Correct organization of the actin cytoskeleton allows sarcomeres to assemble in an orderly manner, conferring mechanical resistance and functional viability to muscle fibers [115]. These findings demonstrate that both GO:BPs can directly influence muscle development and mass, reflecting in alterations of traits such as REA. Among the genes enriched for actin filament bundle organization, FAM107 stands out. By modulating myofibril structure and fiber stability, FAM107A can influence skeletal muscle growth and mass [116]. Thus, variations in this gene may directly affect traits associated with muscle development.

Regarding SFT, several GO:BPs were also consistently identified across GWAS approaches. Notably, lipid homeostasis (GO:0055088) refers to the balance between lipid synthesis, storage, and degradation within cells [117] A study conducted by [118], evaluating fatty acid (FA) composition in different adipose tissue depots in cattle and the expression of genes related to lipid metabolism, identified significant variations both in FA content and gene expression among the different adipose tissues, suggesting that lipid homeostasis can be modulated according to fat depot. According to [119], the molecular regulation of adipogenesis and lipogenesis, mediated by transcription factors such as PPARγ and C/EBPα, controls adipocyte differentiation and lipid synthesis, central processes for lipid homeostasis and adipose tissue expansion, affecting SFT. Another GO: BP of note was the lipid metabolic process (GO:0019216). Evidence from cattle studies indicates that variations in genes associated with this process, such as *ELOVL6*, play an important role in body fat deposition. This gene modulates PPARγ expression and alters fatty acid composition, thereby directly influencing adipocyte proliferation and the regulation of essential lipid pathways. Consequently, alterations in ELOVL6 contribute to differences in subcutaneous fat deposition and, thus, to the expression of SFT in cattle [120].

The most enriched gene for both SFT GO:BPs was *ORMDL*. This gene regulates ceramide synthesis, a bioactive group of lipids that influences insulin resistance, inflammation, and adipocyte expansion. ORMDL proteins inhibit serine palmitoyl transferase (SPT) in response to elevated ceramide levels, maintaining sphingolipid homeostasis [121]. When *ORMDL1* is dysregulated, ceramide levels increase, promoting adipose tissue dysfunction and contributing to insulin resistance and inflammation [122,123]. Regulation of this gene over ceramide synthesis affects adipocyte expansion and function, modulating the capacity of adipose tissue to accumulate lipids, which may impact SFT.

Regarding Cellular Components in REA, two terms stand out: stress fiber (GO:0001725) and actomyosin (GO:0042641). Both represent cellular structures directly associated with contraction and skeletal muscle development [124]. Stress fibers are bundles of actin and myosin that provide mechanical support and participate in force generation, contributing to muscle fiber growth and stability. Actomyosin corresponds to the molecular complex responsible for muscle contraction, formed by the interaction between actin and myosin, and is fundamental for muscle hypertrophy. A transcriptomic study in Simmental cattle showed that phosphorylation of the Alpha-actin 1 protein reduces its affinity for actin, contributing to stress fiber formation. Additionally, the authors reported that the actin-myosin interaction (actomyosin), when not separated by ATP action, causes stiffening of muscle fibers, highlighting its role in muscle structural stability [125]. These mechanisms corroborate the relevance of these GO:CCs in regulating the arrangement of skeletal fibers and help explain their recurrent detection in REA results.

Among the genes enriched for both GO:CC terms, *PDLIM3* stands out. This gene encodes a protein with PDZ and LIM domains, both of which can mediate interactions with proteins essential for cytoskeletal organization. This gene is highly expressed in skeletal muscle, and its protein is specifically localized at the Z-line, where it binds to α-actinin-2 and assists in the organization and anchoring of contractile filaments [126]. Additionally, studies conducted in mice suggested that depletion of *ALP* genes, such as *PDLIM3*, can impair Z-line renewal, causing myofibril disorganization and loss of muscle fiber integrity [127,128]. For these reasons, this gene may affect muscle development and, consequently, REA values.

For SFT, through GO:CC enrichment, several terms were consistently identified across GWAS approaches, among which two stand out: peroxisome (GO:0005777) and endosome (GO:0005768), as they reveal structures directly related to lipid metabolism. The presence of these terms reinforces their functional consistency and suggests that these organelles play fundamental roles in modulating SFT. Peroxisomes are key elements in cellular lipid homeostasis, acting in the β-oxidation of very long and branched-chain fatty acids, and participating in the modulation of sterol metabolism [129,130]. Thus, they contribute to the regulation of intracellular lipid flux and the availability of metabolic intermediates used in lipid synthesis and storage pathways [131]. Endosomes are essential organelles for the trafficking and recycling of membrane receptors, including those involved in insulin signaling, such as the Insulin Receptor and Glucose Transporter Type 4 [132,133,134]. Subcutaneous fat deposition strongly depends on insulin sensitivity and glucose uptake by adipocytes; alterations in endosomal processes can directly modulate the rate of lipogenesis and consequently influence SFT. Among the genes associated with the peroxisome term, *ACOX2* stands out, which was also detected in the KEGG PPAR signaling pathway and similarly highlighted in association with SFT, reinforcing the hypothesis of its influence on the trait, as it was recurrently detected across GWAS approaches and enriched by two distinct approaches. For the GO:CC endosome, the recurrently enriched gene was *PXK*, which encodes a protein localized to endosomes and responsible for accelerating the internalization and trafficking of membrane receptors [135]. This function suggests that variants in this gene may modulate insulin sensitivity dependent on receptor endocytosis and recycling, consequently influencing subcutaneous fat deposition.

The GO enrichment for Molecular Functions in REA identified several terms recurrent across the evaluated GWAS approaches, some of which stand out for their direct involvement in the structural and contractile regulation of skeletal muscle, forming the molecular basis of muscle hypertrophy. Among them, the term calcium/calmodulin-dependent protein kinase activity (CaMK) (GO:0004683) is notable for its physiological relevance in the central role of Ca^2+^/calmodulin signaling in regulating skeletal muscle development and hypertrophy [136]. This function is supported by evidence showing that CaMKs promote the phosphorylation of class II Histone Deacetylases, releasing the activation of Myocyte Enhancer Factor 2-dependent genes, which are important for the differentiation and maturation of muscle fibers [137]. Thus, the recurrence of this term across GWAS approaches suggests that alterations in CaMK activity may influence the phenotypic performance of REA. Another GO:MF to highlight is actin binding (GO:0003779), which groups proteins that bind and regulate actin filaments. These functions are essential for sarcomere assembly, stability, and dynamics [138]. According to [139], since actin is a fundamental component of cytoskeletal dynamics, actin-binding proteins become essential factors for skeletal muscle health, and their dysregulation can lead to muscle atrophy and loss of mass. A transcriptomic study conducted in the longissimus dorsi muscle of cattle demonstrated that genes associated with actin binding were enriched in animals with differences in muscle fiber arrangement development [140]. These studies indicate that actin-associated functions are relevant for muscle metabolism and contribute to their possible relationship with REA.

The gene enriched for the GO:MF CaMK was *CAMK2B*. A study in goats demonstrated that this gene encodes a subunit of CaMKII kinase, whose expression regulates the proliferation and differentiation of skeletal muscle satellite cells [141]. Furthermore, a study in cattle conducted by [142], showed that miR-24-3p regulates *CAMK2B* and modulates proliferation and apoptosis in muscle satellite cells, suggesting that genetic or epigenetic variations in this gene may directly impact phenotypes related to muscle mass. This indicates that *CAMK2B*, through key molecular mechanisms, may contribute to the phenotypic variation observed for REA. The gene *MYO1G* appears to be recurrently associated with GO: MF actin binding. This gene encodes short-tail myosin I, expressed in B and T lymphocytes and macrophages, regulating adhesion, migration, and phagocytosis, and is also important for antigen detection [143]. The association of *MYO1G* is likely indirect and possibly mediated by immune system effects on muscle tissue.

For SFT, GO:MF term enrichment revealed results consistent with the physiology of subcutaneous fat deposition. Among the most relevant terms, lipid binding (GO:0008289) stood out, being identified by all evaluated GWAS approaches, suggesting its strong relationship with the studied phenotype. According to [144], specific proteins that can reversibly and non-covalently associate with lipids, called lipid binding proteins or lipid chaperones, significantly increase the aqueous solubility of lipids, facilitating their transport between tissues and within cells. This results in increased transport and intracellular availability of fatty acids, favoring triglyceride accumulation in adipocytes and directly contributing to subcutaneous fat deposition. A GWAS study in Nellore cattle also found lipid binding GO:MF enriched for the SFT trait [73]. These findings reinforce the association of this GO:MF with the studied trait. Another relevant GO:MF enriched for SFT identified in our study was growth factor activity (GO:0008083). This GO:MF is directly related to fat deposition, as numerous growth factors act as important regulators of adipogenesis, controlling the proliferation and differentiation of pre-adipocytes and promoting lipid accumulation in adipose tissue [145,146,147].

The gene enriched for the GO:MF lipid binding, recurrently detected across GWAS approaches, was *ANXA4*. A study in humans demonstrated that this gene is associated with lipid profiles and lipid metabolites [148]. This suggests that the action of this gene may be related to fat deposition. For the GO:MF growth factor activity, the enriched gene recurrent across GWAS approaches was *MSTN*. This gene is associated with SFT, but in an antagonistic manner, as it promotes myostatin expression and reduces adipogenesis, increasing muscle growth while decreasing the energy available for lipid storage [149]. A study conducted in Marchigiana cattle demonstrated that the loss of *MSTN* function promoted increased muscle mass in the animals and a reduction in subcutaneous and intramuscular fat deposition [150]. These findings support the potential influence of this gene on SFT values.

Overall, the results from KEGG, MeSH, and GO enrichment analyses converge on functional mechanisms directly associated with the physiology of REA and SFT, suggesting that the genomic regions identified by the different GWAS approaches reflect muscular and metabolic processes essential to the expression of these phenotypes. The recurrence of pathways, terms, and functions across the different approaches reinforces the functional robustness of the identified regions, indicating that, despite statistical differences among approaches, there is a common core of mechanisms involved in muscle hypertrophy, insulin sensitivity, adipogenesis, and lipid metabolism. Thus, integrating these results demonstrates that combining GWAS approaches with enrichment analyses provides a concise overview of the physiological processes that modulate REA and SFT in beef cattle.

## 5. Conclusions

The use of different weighted GWAS approaches increased sensitivity in detecting significant windows associated with REA and SFT compared to UM. The QM and A_1.5 approach stand out for identifying more relevant regions. However, this greater sensitivity requires caution, as it may generate inflated signals, especially in populations with small effective population size and for polygenic traits. Extreme weighting approaches may also compromise the quality of associations due to false negatives or low-resolution GWAS. Additionally, the sample size of the genotyped population limits the ability to distinguish the effects of highly correlated SNPs in a context of strong LD, increasing the instability of weighted methods. Consistently, all approaches identified a central set of windows, indicating robust regions associated with REA and SFT. The occurrence of repeated encircled genes in the selected windows suggests that they have fundamental roles in muscle development and fat deposition. Moreover, the multiple ocurrence of KEGG pathways and GO and MeSH terms suggests that those terms are essential for lipid metabolism, cellular signaling, and muscle growth. Therefore, combining different GWAS strategies increases the discovery power and reinforces biologically relevant signals, improving the interpretation of regions, genes, and pathways associated with carcass composition.

## Figures and Tables

**Table 1:** Descriptive statistics, variance components, and heritability for REA and SFT.

Trait	N	Mean	SD	Nº GC	NAG	$\sigma_{a}^{2}$	$\sigma_{e}^{2}$	h^2^ ± SE
REA	2697	63.69	12.56	64	1405	16.837	48.802	0.26 ± 0.05
SFT	2141	3.37	2.11	64	1405	0.555	1.943	0.22 ± 0.04

N: number of animals; CG: contemporary group number; $\sigma_{a}^{2}$: additive genetic; $\sigma_{e}^{2}$: residual variance; h^2^ ± SD= estimate of heritability and standard deviation; NAG: number of animals genotyped.

## Data Availability

The phenotypic and genomic data used in this study are a property of the industry partner that contributed to the study and therefore are not readily available due to its commercially sensitivity.

## Supplementary Materials

The following supporting information can be downloaded at https://www.mdpi.com/article/10.3390/genes17040385/s1: Table S1: Genomic windows of 20 adjacent SNPs explaining 0.5% or more of the AGV, identified across five GWAS approaches for REA; Table S2: Genomic windows of 20 adjacent SNPs explaining 0.5% or more of the AGV for SFT, identified across five GWAS approaches for SFT; Table S3: Genes distributed in genomic windows explained more than 0.5% of additive genetics for the Ribeye Area on the different GWAS methods; Table S4: KEGG functional enrichment related to the Ribeye Area (REA) trait; Table S5: KEGG functional enrichment related to the Subcutaneous Fat Thickness (SFT) trait; Table S6: Enriched MeSH terms related to the Ribeye Area (REA) trait; Table S7: Enriched MeSH terms related to the Subcutaneous Fat Thickness (SFT) trait; Table S8: GO Enrichment analysis for biological process for Ribeye Area (REA) trait; Table S9: GO Enrichment analysis for biological process for Fat Thickness (SFT) trait; Table S10: GO Enrichment analysis for cellular component process for Ribeye Area (REA) trait; Table S11: GO Enrichment analysis for cellular component process for Fat Thickness (SFT) trait; Table S12: GO Enrichment analysis for molecular function component process for Ribeye Area (REA) trait; Table S13: GO Enrichment analysis for molecular function for Fat Thickness (SFT) trait.

## Abbreviations

AGV	Additive Genetic Variance
ATP	Adenosine Triphosphate
BLUP	Best Linear Unbiased Prediction
CaMK	Calcium/Calmodulin-Dependent Protein Kinase
CG	Contemporary Group
CPT1B	Carnitine Palmitoyltransferase 1B
FA	Fatty Acid
GBLUP	Genomic Best Linear Unbiased Prediction
GEBV	Genomic Estimated Breeding Value
GO	Gene Ontology
GO:BP	Gene Ontology Biological Process
GO:CC	Gene Ontology Cellular Component
GO:MF	Gene Ontology Molecular Function
GWAS	Genome-Wide Association Study
GVA	Genetic Variance Explained
h^2^	Heritability
IGF	Insulin-like Growth Factor
KEGG	Kyoto Encyclopedia of Genes and Genomes
LD	Linkage Disequilibrium
MAF	Minor Allele Frequency
MeSH	Medical Subject Headings
NAG	Number of Animals Genotyped
Ne	Effective Population Size
QM	Quadratic Method
QTL	Quantitative Trait Loci
REA	Ribeye Area
REML	Restricted Maximum Likelihood
SD	Standard Deviation
SE	Standard Error
SFT	Subcutaneous Fat Thickness
SNP	Single Nucleotide Polymorphism
SPT	Serine Palmitoyl Transferase
ssGBLUP	Single-Step Genomic Best Linear Unbiased Prediction
ssGWAS	Single-Step Genome-Wide Association Study
UM	Unweighted Method
A_1.125	Non-Linear A (k = 1.125)
A_1.2	Non-Linear A (k = 1.2)
A_1.5	Non-Linear A (k = 1.5)

## References

[1] Goddard M.E., Hayes B.J., Meuwissen T.H.E. (2010). Genomic Selection in Livestock Populations. Genet. Res..

[2] da Silva Neto J.B., Peripoli E., Pereira A.S.C., Stafuzza N.B., Lôbo R.B., Fukumasu H., Ferraz J.B.S., Baldi F. (2023). Weighted Genomic Prediction for Growth and Carcass-Related Traits in Nelore Cattle. Anim. Genet..

[3] Rojas De Oliveira H., Ventura H.T., Costa E.V., Pereira M.A., Veroneze R., Duarte M.D.S., Dias De Siqueira O.H.G.B., Fonseca E Silva F. (2018). Meta-Analysis of Genetic-Parameter Estimates for Reproduction, Growth and Carcass Traits in Nellore Cattle by Using a Random-Effects Model. Anim. Prod. Sci..

[4] Silva-Vignato B., Coutinho L.L., Poleti M.D., Cesar A.S.M., Moncau C.T., Regitano L.C.A., Balieiro J.C.C. (2019). Gene Co-Expression Networks Associated with Carcass Traits Reveal New Pathways for Muscle and Fat Deposition in Nelore Cattle 06 Biological Sciences 0604 Genetics. BMC Genom..

[5] Hayes B., Goddard M. (2010). Genome-Wide Association and Genomic Selection in Animal Breeding. Genome.

[6] VanRaden P.M. (2008). Efficient Methods to Compute Genomic Predictions. J. Dairy Sci..

[7] Misztal I., Aggrey S.E., Muir W.M. (2013). Experiences with a Single-Step Genome Evaluation. Poult. Sci..

[8] Aguilar I., Misztal I., Johnson D.L., Legarra A., Tsuruta S., Lawlor T.J. (2010). Hot Topic: A Unified Approach to Utilize Phenotypic, Full Pedigree, and Genomic Information for Genetic Evaluation of Holstein Final Score. J. Dairy Sci..

[9] Wang H., Misztal I., Aguilar I., Legarra A., Muir W.M. (2012). Genome-Wide Association Mapping Including Phenotypes from Relatives without Genotypes. Genet. Res..

[10] Fragomeni B.O., Lourenco D.A.L., Legarra A., VanRaden P.M., Misztal I. (2019). Alternative SNP Weighting for Single-Step Genomic Best Linear Unbiased Predictor Evaluation of Stature in US Holsteins in the Presence of Selected Sequence Variants. J. Dairy Sci..

[11] Lourenco D.A.L., Fragomeni B.O., Bradford H.L., Menezes I.R., Ferraz J.B.S., Aguilar I., Tsuruta S., Misztal I. (2017). Implications of SNP Weighting on Single-Step Genomic Predictions for Different Reference Population Sizes. J. Anim. Breed. Genet..

[12] Santana B.F., Riser M., Hay E.H.A., Fragomeni B.d.O. (2023). Alternative SNP Weighting for Multi-Step and Single-Step Genomic BLUP in the Presence of Causative Variants. J. Anim. Breed. Genet..

[13] Zhang X., Lourenco D., Aguilar I., Legarra A., Misztal I. (2016). Weighting Strategies for Single-Step Genomic BLUP: An Iterative Approach for Accurate Calculation of GEBV and GWAS. Front. Genet..

[14] Misztal I., Tsuruta S., Lourenco D., Aguilar I., Legarra A., Vitezica Z. (2014). Manual for BLUPF90 Family of Programs.

[15] Harville D.A. (1977). Maximum Likelihood Approaches to Variance Component Estimation and to Related Problems. J. Am. Stat. Assoc..

[16] Legarra A., Aguilar I., Misztal I. (2009). A Relationship Matrix Including Full Pedigree and Genomic Information. J. Dairy Sci..

[17] Espigolan R., Baldi F., Boligon A.A., Souza F.R.P., Gordo D.G.M., Tonussi R.L., Cardoso D.F., Oliveira H.N., Tonhati H., Sargolzaei M. (2013). Study of Whole Genome Linkage Disequilibrium in Nellore Cattle. BMC Genom..

[18] Fragomeni B.d.O., Misztal I., Lourenco D.L., Aguilar I., Okimoto R., Muir W.M. (2014). Changes in Variance Explained by Top SNP Windows over Generations for Three Traits in Broiler Chicken. Front. Genet..

[19] Zhuang Z., Xu L., Yang J., Gao H., Zhang L., Gao X., Li J., Zhu B. (2020). Weighted Single-Step Genome-Wide Association Study for Growth Traits in Chinese Simmental Beef Cattle. Genes.

[20] Dodd G.R., Gray K., Huang Y., Fragomeni B. (2022). Single-Step GBLUP and GWAS Analyses Suggests Implementation of Unweighted Two Trait Approach for Heat Stress in Swine. Animals.

[21] Strandén I., Garrick D.J. (2009). Derivation of Equivalent Computing Algorithms for Genomic Predictions and Reliabilities of Animal Merit. J. Dairy Sci..

[22] Cole J.B., van Raden P.M., O’Connell J.R., van Tassell C.P., Sonstegard T.S., Schnabel R.D., Taylor J.F., Wiggans G.R. (2009). Distribution and Location of Genetic Effects for Dairy Traits. J. Dairy Sci..

[23] Aguilar I., Legarra A., Cardoso F., Masuda Y., Lourenco D., Misztal I. (2019). Frequentist *p*-values for large-scale-single step genome-wide association, with an application to birth weight in American Angus cattle. Genet. Sel. Evol..

[24] Fonseca P.A.S., Suárez-Vega A., Marras G., Cánovas Á. (2020). GALLO: An R Package for Genomic Annotation and Integration of Multiple Data Sources in Livestock for Positional Candidate Loci. Gigascience.

[25] Yu G., Wang L.G., Han Y., He Q.Y. (2012). ClusterProfiler: An R Package for Comparing Biological Themes among Gene Clusters. OMICS.

[26] Wickham H., Vaughan D., Girlich M. tidyr: Tidy messy data; R package version 1.3.2, 2023. https://cran.r-project.org/package=tidyr.

[27] Mailund T. (2019). Manipulating Data Frames: Dplyr. R Data Science Quick Reference: A Pocket Guide to APIs, Libraries, and Packages.

[28] Pereira L.S., Brunes L.C., Baldi F., do Carmo A.S., Soares B.B., Magnabosco V., da Costa Eifert E., Magnabosco C.U. (2023). Genetic Association between Feed Efficiency, Growth, Scrotal Circumference, and Carcass Traits in Guzerat Cattle. Trop. Anim. Health Prod..

[29] Cancino-Baier D.E., Mamani G.C., Santana B.F., Mattos E.C., Eler J.P., Sainz R.D., Tonetto T., Tonetto V., Tonetto F., Quiñones J.A. (2019). Research Article Estimation of Variance Components for Carcass and Production Traits in Guzerat Cattle. Genet. Mol. Res..

[30] Bessa A.F.d.O., Duarte I.N.H., Rola L.D., Bernardes P.A., Gonzaga Neto S., Lôbo R.B., Munari D.P., Buzanskas M.E. (2021). Genetic Evaluation for Reproductive and Productive Traits in Brahman Cattle. Theriogenology.

[31] Freitas T.C., Ventura H.T., e Silva F.F., Veroneze R., Costa E.V., da Silva D.A., Marques D.B.D., Lopes P.S. (2022). Genetic Parameters for Growth, Reproductive, and Carcass Traits in Tabapuã Cattle. Rev. Bras. Zootec..

[32] Moraes G.F., Abreu L.R.A., Toral F.L.B., Ferreira I.C., Ventura H.T., Bergmann J.A.G., Pereira I.G. (2019). Selection for Feed Efficiency Does Not Change the Selection for Growth and Carcass Traits in Nellore Cattle. J. Anim. Breed. Genet..

[33] Bonamy M., Kluska S., Peripolli E., de Lemos M.V.A., Amorim S.T., Vaca R.J., Lôbo R.B., de Castro L.M., de Faria C.U., Borba Ferrari F. (2019). Genetic Association between Different Criteria to Define Sexual Precocious Heifers with Growth, Carcass, Reproductive and Feed Efficiency Indicator Traits in Nellore Cattle Using Genomic Information. J. Anim. Breed. Genet..

[34] De Moraes G.F., Abreu L.R.A., Ferreira I.C., Pereira I.G. (2017). Análise Genética Do Consumo Alimentar Residual Ajustado Para Gordura e de Características de Carcaça e Desempenho Em Um Rebanho Nelore. Ciência Rural.

[35] de Nadai Bonin M., Pedrosa V.B., da Luz e Silva S., Bünger L., Ross D., da Costa Gomes R., de Almeida Santana M.H., de Córdova Cucco D., de Rezende F.M., Ítavo L.C.V. (2021). Genetic Parameters Associated with Meat Quality of Nellore Cattle at Different Anatomical Points of Longissimus: Brazilian Standards. Meat Sci..

[36] Vallejo R.L., Leeds T.D., Gao G., Parsons J.E., Martin K.E., Evenhuis J.P., Fragomeni B.O., Wiens G.D., Palti Y. (2017). Genomic Selection Models Double the Accuracy of Predicted Breeding Values for Bacterial Cold Water Disease Resistance Compared to a Traditional Pedigree-Based Model in Rainbow Trout Aquaculture. Genet. Sel. Evol..

[37] Fragomeni B.O., Lourenco D.A.L., Tsuruta S., Masuda Y., Aguilar I., Legarra A., Lawlor T.J., Misztal I. (2015). Hot Topic: Use of Genomic Recursions in Single-Step Genomic Best Linear Unbiased Predictor (BLUP) with a Large Number of Genotypes. J. Dairy Sci..

[38] Kikuchi N., Moreland E., Homma H., Semenova E.A., Saito M., Larin A.K., Kobatake N., Yusupov R.A., Okamoto T., Nakazato K. (2022). Genes and Weightlifting Performance. Genes.

[39] Yang J., Benyamin B., McEvoy B.P., Gordon S., Henders A.K., Nyholt D.R., Madden P.A., Heath A.C., Martin N.G., Montgomery G.W. (2010). Common SNPs Explain a Large Proportion of the Heritability for Human Height. Nat. Genet..

[40] Okut H., Wu X.L., Rosa G.J., Bauck S., Woodward B.W., Schnabel R.D., Taylor J.F., Gianola D. (2013). Predicting Expected Progeny Difference for Marbling Score in Angus Cattle Using Artificial Neural Networks and Bayesian Regression Models. Genet. Sel. Evol..

[41] Buaban S., Lengnudum K., Boonkum W., Phakdeedindan P. (2022). Genome-Wide Association Study on Milk Production and Somatic Cell Score for Thai Dairy Cattle Using Weighted Single-Step Approach with Random Regression Test-Day Model. J. Dairy Sci..

[42] Legarra A., Lourenco D.A.L., Vitezica Z.G. Bases for Genomic Prediction; 2018. Short Course, Athens, GA, USA. http://genoweb.toulouse.inra.fr/~alegarra/GSIP.pdf.

[43] Brzáková M., Bauer J., Steyn Y., Šplíchal J., Fulínová D. (2022). The Prediction Accuracies of Linear-Type Traits in Czech Holstein Cattle When Using SsGBLUP or WssGBLUP. J. Anim. Sci..

[44] Garcia A.L.S., Bosworth B., Waldbieser G., Misztal I., Tsuruta S., Lourenco D.A.L. (2018). Development of Genomic Predictions for Harvest and Carcass Weight in Channel Catfish 06 Biological Sciences 0604 Genetics. Genet. Sel. Evol..

[45] Mehrban H., Naserkheil M., Lee D.H., Cho C., Choi T., Park M., Ibáñez-escriche N. (2021). Genomic Prediction Using Alternative Strategies of Weighted Single-step Genomic Blup for Yearling Weight and Carcass Traits in Hanwoo Beef Cattle. Genes.

[46] Mancin E., Tuliozi B., Sartori C., Guzzo N., Mantovani R. (2021). Genomic Prediction in Local Breeds: The Rendena Cattle as a Case Study. Animals.

[47] Lopez B.I., Lee S.H., Park J.E., Shin D.H., Oh J.D., Las Heras-Saldana S.D., Van Der Werf J., Chai H.H., Park W., Lim D. (2019). Weighted Genomic Best Linear Unbiased Prediction for Carcass Traits in Hanwoo Cattle. Genes.

[48] Baneh H., Elatkin N., Gentzbittel L. (2025). Genome-Wide Association Studies and Genetic Architecture of Carcass Traits in Angus Beef Cattle Using Imputed Whole-Genome Sequences Data. Genet. Sel. Evol..

[49] Wang Y., Zhang F., Mukiibi R., Chen L., Vinsky M., Plastow G., Basarab J., Stothard P., Li C. (2020). Genetic Architecture of Quantitative Traits in Beef Cattle Revealed by Genome Wide Association Studies of Imputed Whole Genome Sequence Variants: II: Carcass Merit Traits. BMC Genom..

[50] Wittenburg D., Melzer N., Reinsch N. (2011). Including Non-Additive Genetic Effects in Bayesian Methods for the Prediction of Genetic Values Based on Genome-Wide Markers. BMC Genet..

[51] Gianola D., De Los Campos G., Hill W.G., Manfredi E., Fernando R. (2009). Additive Genetic Variability and the Bayesian Alphabet. Genetics.

[52] Filho D.F., de Sousa Bueno Filho J.S., de Almeida Regitano L.C., de Alencar M.M., Alves R.R., Conceição Meirelles S.L. (2019). Tournaments between Markers as a Strategy to Enhance Genomic Predictions. PLoS ONE.

[53] Purfield D.C., Berry D.P., McParland S., Bradley D.G. (2012). Runs of Homozygosity and Population History in Cattle. BMC Genet..

[54] Faria F.J.C., Filho A.E.V., Madalena F.E., Josahkian L.A. (2009). Pedigree Analysis in the Brazilian Zebu Breeds. J. Anim. Breed. Genet..

[55] Santana M.L., Pereira R.J., Bignardi A.B., Ayres D.R., Menezes G.R.O., Silva L.O.C., Leroy G., Machado C.H.C., Josahkian L.A., Albuquerque L.G. (2016). Structure and Genetic Diversity of Brazilian Zebu Cattle Breeds Assessed by Pedigree Analysis. Livest. Sci..

[56] Baldwin-Brown J.G., Long A.D., Thornton K.R. (2014). The Power to Detect Quantitative Trait Loci Using Resequenced, Experimentally Evolved Populations of Diploid, Sexual Organisms. Mol. Biol. Evol..

[57] Meuwissen T., Eikje L.S., Gjuvsland A.B. (2024). GWABLUP: Genome-Wide Association Assisted Best Linear Unbiased Prediction of Genetic Values. Genet. Sel. Evol..

[58] Liu A., Lund M.S., Boichard D., Karaman E., Guldbrandtsen B., Fritz S., Aamand G.P., Nielsen U.S., Sahana G., Wang Y. (2020). Weighted Single-Step Genomic Best Linear Unbiased Prediction Integrating Variants Selected from Sequencing Data by Association and Bioinformatics Analyses. Genet. Sel. Evol..

[59] Casiró S., Velez-Irizarry D., Ernst C.W., Raney N.E., Bates R.O., Charles M.G., Steibel J.P. (2017). Genome-Wide Association Study in an F2 Duroc x Pietrain Resource Population for Economically Important Meat Quality and Carcass Traits. J. Anim. Sci..

[60] Sasazaki S., Yamamoto R., Toyomoto S., Kondo H., Akiyama T., Kohama N., Yoshida E., Kawaguchi F., Oyama K., Mannen H. (2022). Verification of Candidate SNP Effects Reveals Two QTLs on BTA7 for Beef Marbling in Two Japanese Black Cattle Populations. Genes.

[61] Liu W., Sun C., Gao H., He J., Yu A., Xie Y., Yao H., Hu J., Lei Z. (2025). Molecular Regulatory Mechanisms of Dietary Supplementation with Allium Mongolicum Regel Powder to Improve Muscle Development and Meat Quality in Angus Calves. Anim. Biosci..

[62] Widdison S., Ashley G.R., Howard C.J., Coffey T.J. (2007). Characterisation of Bovine Inducible Nitric Oxide Synthase. Vet. Immunol. Immunopathol..

[63] Li R.W., Li C., Gasbarre L.C. (2011). The Vitamin D Receptor and Inducible Nitric Oxide Synthase Associated Pathways in Acquired Resistance to Cooperia Oncophora Infection in Cattle. Vet. Res..

[64] Yu J., Riou C., Davidson D., Minhas R., Robson J.D., Julius M., Arnold R., Kiefer F., Veillette A. (2001). Synergistic Regulation of Immunoreceptor Signaling by SLP-76-Related Adaptor Clnk and Serine/Threonine Protein Kinase HPK-1. Mol. Cell. Biol..

[65] Maier V.K., Feeney C.M., Taylor J.E., Creech A.L., Qiao J.W., Szanto A., Das P.P., Chevrier N., Cifuentes-Rojas C., Orkin S.H. (2015). Functional Proteomic Analysis of Repressive Histone Methyltransferase Complexes Reveals ZNF518B as a G9A Regulator. Mol. Cell. Proteom..

[66] Han S.-H., Cho I.-C., Ko M.-S., Kim E.-Y., Park S.-P., Lee S.-S., Oh H.-S. (2012). A Promoter Polymorphism of MSTN g.- 371T> A and Its Associations with Carcass Traits in Korean Cattle. Mol. Biol. Rep..

[67] Pei Y., Song Y., Feng Z., Li H., Mu Y., Ur Rehman S., Liu Q., Li K. (2022). Myostatin Alteration in Pigs Enhances the Deposition of Long-Chain Unsaturated Fatty Acids in Subcutaneous Fat. Foods.

[68] Niture S., Gyamfi M.A., Lin M., Chimeh U., Dong X., Zheng W., Moore J., Kumar D. (2020). TNFAIP8 Regulates Autophagy, Cell Steatosis, and Promotes Hepatocellular Carcinoma Cell Proliferation. Cell Death Dis..

[69] DeForest N., Wang Y., Zhu Z., Dron J.S., Koesterer R., Natarajan P., Flannick J., Amariuta T., Peloso G.M., Majithia A.R. (2024). Genome-Wide Discovery and Integrative Genomic Characterization of Insulin Resistance Loci Using Serum Triglycerides to HDL-Cholesterol Ratio as a Proxy. Nat. Commun..

[70] Lee C., Eldridge M.J.G., Gonzalez-Lozano M.A., Bresnahan T., Niday Z., Del Camino D., Fu T., Paulo J.A., Moran M.M., Helaine S. (2025). DMXL1 Promotes Recruitment of V1-ATPase to Lysosomes upon TRPML1 Activation. Nat. Struct. Mol. Biol..

[71] Eaton A.F., Danielson E.C., Capen D., Merkulova M., Brown D. (2024). Dmxl1 Is an Essential Mammalian Gene That Is Required for V-ATPase Assembly and Function In Vivo. Function.

[72] Stafuzza N.B., Silva R.M.D.O., Fragomeni B.D.O., Masuda Y., Huang Y., Gray K., Lourenco D.A.L. (2019). A Genome-Wide Single Nucleotide Polymorphism and Copy Number Variation Analysis for Number of Piglets Born Alive. BMC Genom..

[73] Dos Reis H.B., Carvalho M.E., Espigolan R., Poleti M.D., Ambrizi D.R., Berton M.P., Ferraz J.B.S., de Mattos Oliveira E.C., Eler J.P. (2024). Genome-Wide Association (GWAS) Applied to Carcass and Meat Traits of Nellore Cattle. Metabolites.

[74] Silva-Vignato B., Coutinho L.L., Cesar A.S.M., Poleti M.D., Regitano L.C.A., Balieiro J.C.C. (2017). Comparative Muscle Transcriptome Associated with Carcass Traits of Nellore Cattle. BMC Genom..

[75] Arikawa L.M., Mota L.F.M., Schmidt P.I., Frezarim G.B., Fonseca L.F.S., Magalhães A.F.B., Silva D.A., Carvalheiro R., Chardulo L.A.L., Albuquerque L.G. (2024). de Genome-Wide Scans Identify Biological and Metabolic Pathways Regulating Carcass and Meat Quality Traits in Beef Cattle. Meat Sci..

[76] Vallejo R.L., Cheng H., Fragomeni B.O., Shewbridge K.L., Gao G., MacMillan J.R., Towner R., Palti Y. (2019). Genome-Wide Association Analysis and Accuracy of Genome-Enabled Breeding Value Predictions for Resistance to Infectious Hematopoietic Necrosis Virus in a Commercial Rainbow Trout Breeding Population. Genet. Sel. Evol..

[77] Campbell N.R., LaPatra S.E., Overturf K., Towner R., Narum S.R. (2014). Association Mapping of Disease Resistance Traits in Rainbow Trout Using Restriction Site Associated DNA Sequencing. G3 Genes Genomes Genet..

[78] Goddard M. (2009). Genomic Selection: Prediction of Accuracy and Maximisation of Long Term Response. Genetica.

[79] Daetwyler H.D., Pong-Wong R., Villanueva B., Woolliams J.A. (2010). The Impact of Genetic Architecture on Genome-Wide Evaluation Methods. Genetics.

[80] Peixoto M.G.C.D., Carvalho M.R.S., Egito A.A., Steinberg R.S., Bruneli F.Â.T., Machado M.A., Santos F.C., Rosse I.C., Fonseca P.A.S. (2021). Genetic Diversity and Population Genetic Structure of a Guzerá (*Bos Indicus*) Meta-Population. Animals.

[81] Arias J.A., Keehan M., Fisher P., Coppieters W., Spelman R. (2009). A High Density Linkage Map of the Bovine Genome. BMC Genet..

[82] Pocrnic I., Lourenco D.A.L., Masuda Y., Legarra A., Misztal I. (2016). The Dimensionality of Genomic Information and Its Effect on Genomic Prediction. Genetics.

[83] Bodine S.C., Stitt T.N., Gonzalez M., Kline W.O., Stover G.L., Bauerlein R., Zlotchenko E., Scrimgeour A., Lawrence J.C., Glass D.J. (2001). Akt/MTOR Pathway Is a Crucial Regulator of Skeletal Muscle Hypertrophy and Can Prevent Muscle Atrophy in Vivo. Nat. Cell Biol..

[84] Yoon M.S. (2017). MTOR as a Key Regulator in Maintaining Skeletal Muscle Mass. Front. Physiol..

[85] Zhao Y., Xia X., Wang Q., Hu D., Zhang L., Li X., Ding X., Guo H., Guo Y. (2023). Myostatin Mutation Enhances Bovine Myogenic Differentiation through PI3K/AKT/MTOR Signalling via Removing DNA Methylation of RACK1. Cells.

[86] Baeza M.C., Corva P.M., Soria L.A., Rincon G., Medrano J.F., Pavan E., Villarreal E.L., Schor A., Melucci L., Mezzadra C. (2011). Genetic markers of body composition and carcass quality in grazing Brangus steers. Genet. Mol. Res..

[87] Curi R.A., De Oliveira H.N., Silveira A.C., Lopes C.R. (2005). Effects of Polymorphic Microsatellites in the Regulatory Region of IGF1 and GHR on Growth and Carcass Traits in Beef Cattle. Anim. Genet..

[88] Ge X., Yu J., Jiang H. (2012). Growth Hormone Stimulates Protein Synthesis in Bovine Skeletal Muscle Cells without Altering Insulin-like Growth Factor-I MRNA Expression. J. Anim. Sci..

[89] Jiang H., Ge X. (2014). Meat Science and Muscle Biology Symposium-Mechanism of Growth Hormone Stimulation of Skeletal Muscle Growth in Cattle. J. Anim. Sci..

[90] Di Stasio L., Destefanis G., Brugiapaglia A., Albera A., Rolando A. (2005). Polymorphism of the GHR Gene in Cattle and Relationships with Meat Production and Quality. Anim. Genet..

[91] Andrade M.L., Gilio G.R., Perandini L.A., Peixoto A.S., Moreno M.F., Castro É., Oliveira T.E., Vieira T.S., Ortiz-Silva M., Thomazelli C.A. (2021). PPARγ-Induced Upregulation of Subcutaneous Fat Adiponectin Secretion, Glyceroneogenesis and BCAA Oxidation Requires MTORC1 Activity. Biochim. Biophys. Acta Mol. Cell Biol. Lipids.

[92] Guo P.P., Yao X.R., Xu Y.N., Jin X., Li Q., Yan C.G., Kim N.H., Li X.Z. (2024). Insulin Interacts with PPARγ Agonists to Promote Bovine Adipocyte Differentiation. Domest. Anim. Endocrinol..

[93] Wang S., Liu T., Peng P., Fu Y., Shi S., Liang S., Chen X., Wang K., Zhou R. (2025). Integrated Transcriptomic Analysis of Liver and Muscle Tissues Reveals Candidate Genes and Pathways Regulating Intramuscular Fat Deposition in Beef Cattle. Animals.

[94] Qiao K., Jiang R., Contreras G.A., Xie L., Pascottini O.B., Opsomer G., Dong Q. (2024). The Complex Interplay of Insulin Resistance and Metabolic Inflammation in Transition Dairy Cows. Animals.

[95] Dos Santos Silva D.B., Fonseca L.F.S., Pinheiro D.G., Muniz M.M.M., Magalhães A.F.B., Baldi F., Ferro J.A., Chardulo L.A.L., De Albuquerque L.G. (2019). Prediction of Hub Genes Associated with Intramuscular Fat Content in Nelore Cattle. BMC Genom..

[96] Du L., Chang T., An B., Liang M., Deng T., Li K., Cao S., Du Y., Gao X., Xu L. (2023). Transcriptomics and Lipid Metabolomics Analysis of Subcutaneous, Visceral, and Abdominal Adipose Tissues of Beef Cattle. Genes.

[97] Reis I.A., Baldassini W.A., Ramírez-Zamudio G.D., de Farias I.M.S.C., Chiaratti M.R., Pereira Junior S., Nociti R.P., Carvalho P.H.V., Curi R.A., Pereira G.L. (2024). Muscle Tissue Transcriptome of F1 Angus-Nellore Bulls and Steers Feedlot Finished: Impacts on Intramuscular Fat Deposition. BMC Genom..

[98] Buse M.G. (2006). Hexosamines, Insulin Resistance, and the Complications of Diabetes: Current Status. Am. J. Physiol.-Endocrinol. Metab..

[99] Lee S.H., Park S.Y., Choi C.S. (2022). Insulin Resistance: From Mechanisms to Therapeutic Strategies. Diabetes Metab. J..

[100] Teo C.F., Wollaston-Hayden E.E., Wells L. (2010). Hexosamine Flux, the O-GlcNAc Modification, and the Development of Insulin Resistance in Adipocytes. Mol. Cell. Endocrinol..

[101] Morota G., Peñagaricano F., Petersen J.L., Ciobanu D.C., Tsuyuzaki K., Nikaido I. (2015). An Application of MeSH Enrichment Analysis in Livestock. Anim. Genet..

[102] Meister A. (2012). Biochemistry of the Amino Acids.

[103] Kim J., Kim W.S. (2023). Arginine and Lysine Promote Skeletal Muscle Hypertrophy by Regulating the MTOR Signaling Pathway in Bovine Myocytes. Meat Muscle Biol..

[104] Li X., Cao Y., Liu Y., Fang W., Xiao C., Zhao Y. (2024). Effect of IGF1 on Myogenic Proliferation and Differentiation of Bovine Skeletal Muscle Satellite Cells Through PI3K/AKT Signaling Pathway. Genes.

[105] Nakatani M., Kokubo M., Ohsawa Y., Sunada Y., Tsuchida K. (2011). Follistatin-Derived Peptide Expression in Muscle Decreases Adipose Tissue Mass and Prevents Hepatic Steatosis. Am. J. Physiol. Endocrinol. Metab..

[106] Amthor H., Nicholas G., McKinnell I., Kemp C.F., Sharma M., Kambadur R., Patel K. (2004). Follistatin Complexes Myostatin and Antagonises Myostatin-Mediated Inhibition of Myogenesis. Dev. Biol..

[107] Singh R., Pervin S., Lee S.J., Kuo A., Grijalva V., David J., Vergnes L., Reddy S.T. (2018). Metabolic Profiling of Follistatin Overexpression: A Novel Therapeutic Strategy for Metabolic Diseases. Diabetes Metab. Syndr. Obes..

[108] Knottnerus S.J.G., Bleeker J.C., Wüst R.C.I., Ferdinandusse S., IJlst L., Wijburg F.A., Wanders R.J.A., Visser G., Houtkooper R.H. (2018). Disorders of Mitochondrial Long-Chain Fatty Acid Oxidation and the Carnitine Shuttle. Rev. Endocr. Metab. Disord..

[109] Hoppel C. (2003). The Role of Carnitine in Normal and Altered Fatty Acid Metabolism. Am. J. Kidney Dis..

[110] He W., Gao M., Yang R., Zhao Z., Mi J., Sun H., Xiao H., Fang X. (2022). The Effect of CPT1B Gene on Lipid Metabolism and Its Polymorphism Analysis in Chinese Simmental Cattle. Anim. Biotechnol..

[111] Chen L., Zhang Y.H., Wang S.P., Zhang Y.H., Huang T., Cai Y.D. (2017). Prediction and Analysis of Essential Genes Using the Enrichments of Gene Ontology and KEGG Pathways. PLoS ONE.

[112] Saxena R., Bishnoi R., Singla D. (2021). Gene Ontology: Application and Importance in Functional Annotation of the Genomic Data. Bioinformatics: Methods and Applications.

[113] Rajan S., Kudryashov D.S., Reisler E. (2023). Actin Bundles Dynamics and Architecture. Biomolecules.

[114] Ono S. (2010). Dynamic Regulation of Sarcomeric Actin Filaments in Striated Muscle. Cytoskeleton.

[115] Wang Z., Grange M., Wagner T., Kho A.L., Gautel M., Raunser S. (2021). The Molecular Basis for Sarcomere Organization in Vertebrate Skeletal Muscle. Cell.

[116] Kretzschmar A., Schülke J.P., Masana M., Dürre K., Müller M.B., Bausch A.R., Rein T. (2018). The Stress-Inducible Protein DRR1 Exerts Distinct Effects on Actin Dynamics. Int. J. Mol. Sci..

[117] Bosma M. (2014). Lipid Homeostasis in Exercise. Drug Discov. Today.

[118] Nawaz A., Zhang J., Meng Y., Sun L., Zhou H., Geng C., Liu H., Jin Y., Ji S. (2024). Fatty Acid Profiles Unveiled: Gene Expression in Yanbian Yellow Cattle Adipose Tissues Offers New Insights into Lipid Metabolism. Arch. Anim. Breed..

[119] Moseti D., Regassa A., Kim W.K. (2016). Molecular Regulation of Adipogenesis and Potential Anti-Adipogenic Bioactive Molecules. Int. J. Mol. Sci..

[120] Junjvlieke Z., Khan R., Mei C., Cheng G., Wang S., Raza S.H.A., Hong J., Wang X., Yang W., Zan L. (2020). Effect of ELOVL6 on the Lipid Metabolism of Bovine Adipocytes. Genomics.

[121] Brown R.D.R., Spiegel S. (2023). ORMDL in Metabolic Health and Disease. Pharmacol. Ther..

[122] Clarke B.A., Majumder S., Zhu H., Lee Y.T., Kono M., Li C., Khanna C., Blain H., Schwartz R., Huso V.L. (2019). The *Ormdl* Genes Regulate the Sphingolipid Synthesis Pathway to Ensure Proper Myelination and Neurologic Function in Mice. eLife.

[123] Sokolowska E., Blachnio-Zabielska A. (2019). The Role of Ceramides in Insulin Resistance. Front. Endocrinol..

[124] Pellegrin S., Mellor H. (2007). Actin Stress Fibers. J. Cell Sci..

[125] Du L., Chang T., An B., Liang M., Duan X., Cai W., Zhu B., Gao X., Chen Y., Xu L. (2021). Transcriptome Profiling Analysis of Muscle Tissue Reveals Potential Candidate Genes Affecting Water Holding Capacity in Chinese Simmental Beef Cattle. Sci. Rep..

[126] Klaavuniemi T., Kelloniemi A., Ylänne J. (2004). The ZASP-like Motif in Actinin-Associated LIM Protein Is Required for Interaction with the α-Actinin Rod and for Targeting to the Muscle Z-Line. J. Biol. Chem..

[127] Pashmforoush M., Pomiès P., Peterson K.L., Kubalak S., Ross J., Hefti A., Aebi U., Beckerle M.C., Chien K.R. (2001). Adult Mice Deficient in Actinin–Associated LIM-Domain Protein Reveal a Developmental Pathway for Right Ventricular Cardiomyopathy. Nat. Med..

[128] Zhou Q., Chu P.H., Huang C., Cheng C.F., Martone M.E., Knoll G., Diane Shelton G., Evans S., Chen J. (2001). Ablation of Cypher, a PDZ-LIM Domain Z-Line Protein, Causes a Severe Form of Congenital Myopathy. J. Cell Biol..

[129] Han J., Zheng D., Liu P.S., Wang S., Xie X. (2024). Peroxisomal Homeostasis in Metabolic Diseases and Its Implication in Ferroptosis. Cell Commun. Signal..

[130] Yuan Z.H., Liu T., Wang H., Xue L.X., Wang J.J. (2021). Fatty Acids Metabolism: The Bridge Between Ferroptosis and Ionizing Radiation. Front. Cell Dev. Biol..

[131] Schrader M., Fahimi H.D. (2006). Growth and Division of Peroxisomes. Int. Rev. Cytol..

[132] Lampson M.A., Schmoranzer J., Zeigerer A., Simon S.M., Mcgraw T.E. (2001). Insulin-Regulated Release from the Endosomal Recycling Compartment Is Regulated by Budding of Specialized Vesicles. Mol. Biol. Cell.

[133] Zeigerer A., Lampson M.A., Karylowski O., Sabatini D.D., Adesnik M., Ren M., McGraw T.E. (2002). GLUT4 Retention in Adipocytes Requires Two Intracellular Insulin-Regulated Transport Steps. Mol. Biol. Cell.

[134] O’sullivan M.J., Lindsay A.J. (2020). The Endosomal Recycling Pathway—At the Crossroads of the Cell. Int. J. Mol. Sci..

[135] Takeuchi H., Takeuchi T., Gao J., Cantley L.C., Hirata M. (2010). Characterization of PXK as a Protein Involved in Epidermal Growth Factor Receptor Trafficking. Mol. Cell. Biol..

[136] Chin E.R. (2005). Role of Ca^2+^/Calmodulin-Dependent Kinases in Skeletal Muscle Plasticity. J Appl Physiol.

[137] Bassel-Duby R., Olson E.N. (2006). Signaling Pathways in Skeletal Muscle Remodeling. Annu. Rev. Biochem..

[138] Prill K., Dawson J.F. (2020). Assembly and Maintenance of Sarcomere Thin Filaments and Associated Diseases. Int. J. Mol. Sci..

[139] Nguyen M.T., Dash R., Jeong K., Lee W. (2023). Role of Actin-Binding Proteins in Skeletal Myogenesis. Cells.

[140] Wang S., Raza S.H.A., Mei C., Zhu K., Garcia M., Schreurs N.M., Liang C., Yang X., Zan L. (2020). Transcriptome Profiling Reveals Differential Expression of Genes Potentially Involved in Muscle and Adipose Tissue Development of Cattle. Electron. J. Biotechnol..

[141] Jing J., Zhang S., Wei J., Yang Y., Zheng Q., Zhu C., Li S., Cao H., Fang F., Liu Y. (2023). MiR-188-5p Regulates the Proliferation and Differentiation of Goat Skeletal Muscle Satellite Cells by Targeting Calcium/Calmodulin Dependent Protein Kinase II Beta. Anim. Biosci..

[142] Yang G., Wu M., Liu X., Wang F., Li M., An X., Bai F., Lei C., Dang R. (2022). MiR-24-3p Conservatively Regulates Muscle Cell Proliferation and Apoptosis by Targeting Common Gene CAMK2B in Rat and Cattle. Animals.

[143] Coluccio L.M., Coluccio L.M. (2020). Myosins and Disease. Myosins: A Superfamily of Molecular Motors.

[144] Glatz J.F.C. (2015). Lipids and Lipid Binding Proteins: A Perfect Match. Prostaglandins Leukot. Essent. Fat. Acids.

[145] Hu L., Yang G., Hägg D., Sun G., Ahn J.M., Jiang N., Ricupero C.L., Wu J., Rodhe C.H., Ascherman J.A. (2015). IGF1 Promotes Adipogenesis by a Lineage Bias of Endogenous Adipose Stem/Progenitor Cells. Stem Cells.

[146] Bell L.N., Ward J.L., Degawa-Yamauchi M., Bovenkerk J.E., Jones R., Cacucci B.M., Gupta C.E., Sheridan C., Sheridan K., Shankar S.S. (2006). Adipose Tissue Production of Hepatocyte Growth Factor Contributes to Elevated Serum HGF in Obesity. Am. J. Physiol. Endocrinol. Metab..

[147] Holly J., Perks C., Shield J. (2006). Adipogenesis and IGF-1. Metab. Syndr. Relat. Disord..

[148] Petty L.E., Chen H.-H., Frankel E.G., Zhu W., Downie C.G., Graff M., Lin P., Sharma P., Zhang X., Scartozzi A.C. (2025). Large-Scale Multi-Omics Analyses in Hispanic/Latino Populations Identify Genes for Cardiometabolic Traits. Nat. Commun..

[149] Lin J., Arnold H.B., Della-Fera M.A., Azain M.J., Hartzell D.L., Baile C.A. (2002). Myostatin Knockout in Mice Increases Myogenesis and Decreases Adipogenesis. Biochem. Biophys. Res. Commun..

[150] Ceccobelli S., Perini F., Trombetta M.F., Tavoletti S., Lasagna E., Pasquini M. (2022). Effect of Myostatin Gene Mutation on Slaughtering Performance and Meat Quality in Marchigiana Bulls. Animals.

