# Supplementary material for: Genome Wide Association Studies with Different Weighting Approaches Reveals Genomic Windows Associated with Meat Quality Traits in Beef Cattle
Source: Genes (Basel). 2026 Mar 28;17(4):385. doi: 10.3390/genes17040385 (PMC13116000; doi:10.3390/genes17040385)
Supplement: Supplementary file 1 [file genes-17-00385-s001.zip › Supplementary Materials/Supplementary _Table_ S3_Genes_Distribution.docx]

**Table S3.** Genes distributed in genomic windows explained more than 0.5% of additive genetics for the Ribeye Area on the different GWAS methods

| **GWAS Method** | **Chr** | **Genomic range**  **(start – end position)** | **Gene names** | **%Var** |
| --- | --- | --- | --- | --- |
| **UM** | 2 | 75032049 - 75918514 | *CNTNAP5* | 0.81 |
| **UM** | 4 | 76581603 - 77774235 | *NUDCD3, CAMK2B, PURB, H2AZ2, TBRG4, PPIA, CCM2, MYO1G, IGFBP1, ZMIZ2, OGDH, IGFBP3, RAMP3, ADCY1, TMED4, DDX56,* *NPC1L1, U6, bta-mir-4657* | 0.75 |
|  |  | 80277910 - 81169085 | *SUGCT* | 0.51 |
| **UM** | 6 | 105912944 - 107227062 | *ZNF518B, CLNK* | 0.71 |
| **UM** | 12 | 30852113 - 32681456 | *UBL3, KATNAL1, SNORA70, SLC7A1, MTUS2* | 0.78 |
| **UM** | 16 | 33655657 - 34881990 | *ZBTB18, AKT3, SDCCAG8, CEP170* | 0.78 |
| **UM** | 19 | 19002409 - 19765067 | *LYRM9, WSB1, RAB11FIP4, NF1, EVI2A, EVI2B, OMG, KSR1, LGALS9, NOS2* | 0.80 |
| **UM** | 20 | 2522258 - 3588633 | *RANBP17, C5orf58, LCP2, KCNIP1, KCNMB1, GABRP, U6, bta-mir-12032* | 0.52 |
| **UM** | 22 | 42593389 - 44047775 | *CFAP20DC, FAM3D, FAM107A, ACOX2, KCTD6, PDHB, RPP14, HTD2, ABHD6, U6, PXK* | 0.84 |
| **UM** | 23 | 46249030 - 47505469 | *-* | 0.65 |
| **QM** | 1 | 13778296 - 15074076 | *-* | 0.71 |
| **QM** | 2 | 75889261 - 77090209 | *CNTNAP5* | 1.35 |
|  |  | 90237664 - 91563786 | *MPP4, ALS2, CDK15, FZD7, KIAA2012, U6* | 0.63 |
| **QM** | 3 | 23560725 - 24815752 | *SEC22B, NOTCH2, REG4, HMGCS2, PHGDH, ZNF697, WARS2, HSD3B1, HAO2, PDE4DIP, U2* | 1.95 |
| **QM** | 4 | 38363077 - 39786318 | *CACNA2D1* | 0.72 |
|  |  | 80277910 - 81169085 | *SUGC* | 1.38 |
|  |  | 81743723 - 82869117 | *RALA, YAE1, VPS41, POU6F2* | 1.59 |
| **QM** | 6 | 105835255 - 107098263 | *ZNF518B, CLNK* | 2.09 |
|  |  | 107226295 - 108591544 | *U6* | 23.53 |
|  |  | 108592440 - 109938102 | *RAB28, NKX3-2, BOD1L1* | 1.61 |
| **QM** | 12 | 9026492 - 10122039 | *-* | 0.61 |
|  |  | 32027380 - 33457798 | *FLT1, bta-mir-2300a, PAN3, FLT3, URAD, CDX2, PDX1, GSX1, POLR1D, LNX2, MTIF3, GTF3A* | 11.83 |
|  |  | 33476176 - 35054878 | *RALA, YAE1, VPS41, POU6F2* | 5.30 |
| **QM** | 16 | 34008163 - 35071667 | *AKT3, SDCCAG8, CEP170, PLD5* | 9.71 |
| **QM** | 16 | 35137739 - 36067442 | *PLD5, BECN2, MAP1LC3C, EXO1, WDR64, CHML, KMO, FH, RGS7* | 1.46 |
| **QM** | 18 | 42411072 - 43284189 | *U6, SNORA70, bta-mir-2899, ZNF507* | 0.64 |
| **QM** | 19 | 19002409 - 19765067 | *LYRM9, NLK, TMEM97, IFT20, TNFAIP1, POLDIP2, TMEM199, SEBOX, VTN, SARM1, SLC46A1, SLC13A2, FOXN1, UNC119, PIGS, ALDOC, SPAG5, RSKR, KIAA0100, SDF2, FLOT2, PHF12, LGALS9, NOS2, SNORA70, SUPT6H, PROCA1, RAB34, RPL23A, TLCD1, NEK8, TRAF4, FAM222B, ERAL1, SNORD42, SNORD42, DHRS13* | 1.00 |
|  |  | 19814327 - 20963068 | *TMEM97, IFT20, TNFAIP1, POLDIP2, TMEM199, SEBOX, VTN, SARM1, SLC46A1, SNORA70, SLC13A2, FOXN1* | 11.13 |
|  |  | 21018421 - 22039421 | *CRYBA1, NUFIP2, TAOK1, ABHD15, TP53I13, GIT1, ANKRD13B, CORO6, SSH2, EFCAB5, NSRP1, SLC6A4, BLMH, TMIGD1, CPD, MYO18A, U6, SNORA72, U6, bta-mir-4523* | 1.18 |
| **QM** | 22 | 43713828 - 45040991 | *FLNB, SLMAP, DENND6A, ARF4, PDE12, DNAH12, ASB14, APPL1, HESX1, IL17RD, ARHGEF3* | 1.5 |
|  |  | 57498869 - 58165788 | *GRIP2, CCDC174, SLC6A6, SYN2, RBSN, MRPS25, NR2C2, FGD5, bta-mir-2888-2, C22H3orf20* | 0.54 |
| **QM** | 23 | 46331376 - 47564094 | *EEF1E1, TXNDC5* | 4.59 |
|  |  | 47625893 - 48714745 | *BMP6, SNRNP48, DSP, SLC35B3, RIOK1, SSR1, RREB1, U6, BLOC1S5* | 1.87 |
| **QM** | 27 | 15236071 - 16259785 | *IRF2, CASP3, PRIMPOL, ACSL1, CENPU, HELT, ENPP6, SLC25A4, CFAP97, SNX25, LRP2BP, ANKRD37, UFSP2, CFAP96, CCDC110, PDLIM3* | 0.82 |
| **A_1.125** | 2 | 75032049 - 75918514 | *-* | 0.86 |
|  |  | 90182742 - 91367222 | *CFLAR, CASP8, FLACC1, TRAK2, STRADB, C2CD6, TMEM237, MPP4, ALS2, CDK15, FZD7, KIAA2012, U6* | 0.55 |
| **A_1.125** | 4 | 76717317 - 77917876 | *NUDCD3, CAMK2B, YKT6, GCK, MYL7, PURB, H2AZ2, TBRG4, PPIA, CCM2, MYO1G, POLD2, AEBP1, ZMIZ2, OGDH, RAMP3, ADCY1, TMED4, DDX56, NPC1L1, U6* | 0.79 |
|  |  | 80277910 - 81169085 | *SUGCT* | 0.69 |
| **A_1.125** | 5 | 52109863 - 53268394 | *TAFA2* | 0.51 |
| **A_1.125** | 6 | 105912944 - 107227062 | *ZNF518B, CLNK* | 1.14 |
| **A_1.125** | 12 | 30852113 - 32681456 | *UBL3, KATNAL1, SNORA70, SLC7A1, MTUS2* | 1.10 |
| **A_1.125** | 16 | 33980423 - 35056607 | *PLD5, AKT3, SDCCAG8, CEP170* | 1.01 |
| **A_1.125** | 19 | 19042090 - 19844499 | *LYRM9, WSB1, RAB11FIP4, NF1, EVI2A, EVI2B, OMG, KSR1, LGALS9, NOS2* | 0.90 |
| **A_1.125** | 20 | 2522258 - 3588633 | *RANBP17, C5orf58, LCP2, KCNIP1, KCNMB1, GABRP, U6, bta-mir-12032* | 0.55 |
| **A_1.125** | 22 | 42593389 - 44047775 | *CFAP20DC, FAM3D, FAM107A, ACOX2, KCTD6, PDHB, RPP14, HTD2, ABHD6, U6, PXK* | 1.08 |
|  |  | 56522913 - 57676680 | *RPL32, CAND2, TMEM40, TMCC1, PLXND1, H1-8, RHO, IFT122, MBD4, RAF1, MKRN2, MKRN2OS, TSEN2, SYN2, TIMP4, bta-mir-11994, bta-mir-2373, bta-mir-2372, EFCAB12* | 0.53 |
| **A_1.125** | 23 | 46331376 - 47564094 | *-* | 0.91 |
| **A_1.2** | 2 | 75032049 - 75918514 | *-* | 0.87 |
|  |  | 90182742 - 91367222 | *CFLAR, CASP8, FLACC1, TRAK2, STRADB, C2CD6, TMEM237, MPP4, ALS2, CDK15, FZD7, KIAA2012, U6* | 0.63 |
| **A_1.2** | 4 | 76717317 - 77917876 | *NUDCD3, CAMK2B, YKT6, GCK, MYL7, PURB, H2AZ2, TBRG4, PPIA, CCM2, MYO1G, POLD2, AEBP1, ZMIZ2, OGDH, RAMP3, ADCY1, TMED4, DDX56, NPC1L1, U6, bta-mir-4657* | 0.79 |
|  |  | 80277910 - 81169085 | *SUGCT* | 0.84 |
| **A_1.2** | 5 | 52109863 - 53268394 | *TAFA2* | 0.58 |
| **A_1.2** | 6 | 105912944 - 107227062 | *U6, ZNF518B, CLNK* | 1.58 |
|  |  | 107300290 - 108668355 | *-* | 0.75 |
| **A_1.2** | 12 | 30852113 - 32681456 | *UBL3, KATNAL1, SNORA70, SLC7A1, MTUS2* | 1.39 |
|  |  | 32788874 - 34171532 | *POLR1D, LNX2, MTIF3, GTF3A, RASL11A, RPL21, USP12, GPR12, CDK8, RNF6, WASF3* | 0.59 |
| **A_1.2** | 16 | 34008163 - 35071667 | *PLD5, AKT3, SDCCAG8, CEP170* | 1.30 |
| **A_1.2** | 19 | 19064643 - 19920352 | *LYRM9, WSB1, RAB11FIP4, NF1, EVI2A, EVI2B, OMG, KSR1, LGALS9, NOS2* | 1.01 |
| **A_1.2** | 20 | 2522258 - 3588633 | *RANBP17, C5orf58, LCP2, KCNIP1, KCNMB1, GABRP, U6, bta-mir-12032* | 0.58 |
| **A_1.2** | 22 | 42593827 - 44235949 | *CFAP20DC, FAM3D, FAM107A, ACOX2, KCTD6, PDHB, RPP14, HTD2, ABHD6, U6, PXK* | 1.27 |
|  |  | 56522913 - 57676680 | *RPL32, CAND2, TMEM40, TMCC1, PLXND1, H1-8, RHO, IFT122, MBD4, RAF1, MKRN2, MKRN2OS, TSEN2, SYN2, TIMP4, bta-mir-11994, bta-mir-2373, bta-mir-2372, EFCAB12* | 0.57 |
| **A_1.2** | 23 | 46331376 - 47564094 | *-* | 1.16 |
| **A_1.2** | 27 | 15236071 - 16259785 | *IRF2, CASP3, PRIMPOL, ACSL1, CENPU, HELT, ENPP6, SLC25A4, CFAP97, SNX25, LRP2BP, ANKRD37, UFSP2, CFAP96, CCDC110, PDLIM3* | 0.51 |
| **A_1.5** | 1 | 13778296 - 15074076 | *-* | 0.80 |
|  |  | 69213596 - 70559726 | *KALRN, UMPS, ITGB5, MUC13, HEG1, SLC12A8, ZNF148* | 1.07 |
|  |  | 70569417 - 72055544 | *MUC4, TNK2, ZDHHC19, SLC51A, PCYT1A, TM4SF19, UBXN7, RNF168, SMCO1, RPL35A, IQCG, FYTTD1, LRCH3, RUBCN, MUC20, U6, TFRC, LMLN* | 0.92 |
|  |  | 98374868 - 99566422 | *MECOM* | 0.57 |
|  |  | 100156848 - 101291051 | *SERPINI1, PDCD10, WDR49, SERPINI2, ZBBX* | 0.88 |
| **A_1.5** | 2 | 75032049 - 75918514 | *-* | 0.69 |
|  |  | 90237664 - 91563786 | *CFLAR, CASP8, FLACC1, TRAK2, STRADB, C2CD6, TMEM237, MPP4, ALS2, CDK15, FZD7, KIAA2012, U6* | 0.64 |
| **A_1.5** | 3 | 22482395 - 23548978 | *BCL9, CHD1L, FMO5, PDE4DIP, OR13L12, U1, U6* | 0.58 |
|  |  | 23560725 - 24815752 | *SEC22B, NOTCH2, REG4, HMGCS2, PHGDH, ZNF697, WARS2, HSD3B1, HAO2, PDE4DIP, U2* | 1.03 |
| **A_1.5** | 4 | 38363077 - 39786318 | *CACNA2D1* | 0.82 |
|  |  | 76717317 - 77917876 | *NUDCD3, CAMK2B, YKT6, GCK, MYL7, PURB, H2AZ2, TBRG4, PPIA, CCM2, MYO1G, POLD2, AEBP1, ZMIZ2, OGDH, RAMP3, ADCY1, TMED4, DDX56, NPC1L1, U6, bta-mir-4657* | 0.57 |
|  |  | 80892637 - 82056167 | *SUGCT, MPLKIP, CDK13, RALA, YAE1, POU6F2* | 1.65 |
| **A_1.5** | 5 | 12704211 - 13494148 | *TMTC2* | 0.64 |
|  |  | 52109863 - 53268394 | *TAFA2* | 0.75 |
| **A_1.5** | 6 | 105912944 - 107227062 | *ZNF518B, CLNK* | 4.93 |
|  |  | 107259582 - 108596491 | *U6* | 3.06 |
|  |  | 108899604 - 110262926 | *RAB28, NKX3-2, BOD1L1* | 0.68 |
| **A_1.5** | 12 | 8972547 - 10024033 | *-* | 1.63 |
|  |  | 31814723 - 33308209 | *ATP8A2, GSX1, POLR1D, SLC46A3, POMP, FLT3, URAD, FLT1, CDX2, PAN3, bta-mir-2300a* | 4.87 |
|  |  | 33418450 - 34711526 | *SHISA2, ATP8A2, NUP58, CDK8, RNF6, WASF3, U6* | 0.96 |
|  |  | 76661176 - 77943237 | *ZIC5, ZIC2, PCCA, U6, GGACT, TMTC4, bta-mir-2892, NALCN, ITGBL1* | 0.55 |
| **A_1.5** | 16 | 34008163 - 35071667 | *PLD5, AKT3, SDCCAG8, CEP170* | 2.66 |
| **A_1.5** | 19 | 19002409 - 19765067 | *LYRM9, WSB1, RAB11FIP4, NF1, EVI2A, EVI2B, OMG, KSR1, LGALS9, NOS2* | 1.05 |
|  |  | 19814327 - 20963068 | *LYRM9, NLK, TMEM97, IFT20, TNFAIP1, POLDIP2, TMEM199, SEBOX, VTN, SARM1, SLC46A1, SLC13A2, FOXN1, UNC119, PIGS, ALDOC, SPAG5, RSKR, KIAA0100, SDF2, FLOT2, PHF12, LGALS9, NOS2, SNORA70, SUPT6H, PROCA1, RAB34, RPL23A, TLCD1, NEK8, TRAF4, FAM222B, ERAL1, SNORD42, SNORD42*, *DHRS13* | 2.04 |
| **A_1.5** | 22 | 42594928 - 44248276 | *CFAP20DC, FAM3D, FAM107A, ACOX2, KCTD6, PDHB, RPP14, HTD2, ABHD6, U6, PXK* | 1.68 |
|  |  | 44527914 - 46346946 | *ARHGEF3, TASOR, CCDC66, ERC2, bta-mir-2371* | 2.11 |
|  |  | 57498869 - 58165788 | *GRIP2, CCDC174, SLC6A6, SYN2, RBSN, MRPS25, NR2C2, FGD5, bta-mir-2888-2, C22H3orf20* | 0.67 |
| **A_1.5** | 23 | 46331376 - 47564094 | *-* | 2.04 |
| **A_1.5** | 27 | 15236071 - 16259785 | *IRF2, CASP3, PRIMPOL, ACSL1, CENPU, HELT, ENPP6, SLC25A4, CFAP97, SNX25, LRP2BP, ANKRD37, UFSP2, CFAP96, CCDC110, PDLIM3* | 0.91 |
| **A_1.5** | 28 | 39266326 - 40484243 | *CDHR1, LRIT2, LRIT1, RGR, CCSER2, GHITM, GPR15LG, U6* | 0.59 |
| **A_1.5** | 29 | 5784033 - 6869558 | *FOLH1, NOX4, U6, U6, U6* | 0.60 |
| Chr = chromosome; %Var = percentage of variance explained by the genomic window; UM = Unweighted method; QM = Quadratic method; A_1.125 = Non-Linear A method with a weighting factor equal to 1.125; A_1.2 = Non-Linear A method with a weighting factor equal to 1.2; A_1.5 = Non-Linear A method with a weighting factor equal to 1.5 | | | | |

**Table S2.** Genes distributed in genomic windows explained more than 0.5% of additive genetics for the Subcutaneous Fat Thickness on the different GWAS methods

| **GWAS Method** | **Chr** | **Genomic range**  **(start – end position)** | **Gene names** | **%Var** |
| --- | --- | --- | --- | --- |
| **UM** | 2 | 6600270-7670949 | *MSTN, PMS1, OSGEPL1, ANKAR, ASNSD1, SLC40A1, WDR75, HIBCH, C2H2orf88, ORMDL1* | 0.58 |
| **UM** | 3 | 115120882-116094718 | *AGAP1, SH3BP4* | 0.51 |
| **UM** | 5 | 7065478-8176883 | *NAV3*, *U6* | 0.68 |
| **UM** | 7 | 34952996-35832365 | *DTWD2, TNFAIP8, DMXL1* | 1.11 |
| **UM** | 11 | 67964661-69208404 | *ANXA4, GMCL1, MXD1, PCBP1, ANTXR1, GFPT1, NFU1, AAK1, ASPRV1* | 0.75 |
| **UM** | 17 | 8641625-9641431 | *IQCM* | 0.71 |
| **UM** | 22 | 42593389-44047775 | *CFAP20DC, FAM3D, FAM107A, ACOX2, KCTD6, PDHB, RPP14, HTD2, ABHD6, U6, PXK* | 0.52 |
| **UM** | 30 | 40874754-43940345 | *-* | 0.57 |
| **QM** | 1 | 131617330 - 133013900 | *CLDN18, SOX14* | 1.55 |
| **QM** | 2 | 6902327 - 7807676 | *COL5A2, COL3A1, PMS1, OSGEPL1, ANKAR, ASNSD1, SLC40A1, WDR75, bta-mir-2917, ORMDL1* | 5.05 |
|  |  | 126497926 - 126976378 | *PDIK1L, PIGV, RPS6KA1, HMGN2, DHDDS, LIN28A, ARID1A, ZNF683, CRYBG2, CD52, UBXN11, SH3BGRL3, CEP85, CATSPER4, CNKSR1, ZNF593OS, FAM110D, ZNF593, C1orf232, KDF1, NUDC, NR0B2, GPATCH3, GPN2, SFN, ZDHHC18, WDTC1, SLC9A1, TENT5B, TRNP1* | 1.82 |
|  |  | 58476871 - 59243122 | *NXPH2, SPOPL* | 1.38 |
|  |  | 7819857 - 9004095 | *COL5A2, COL3A1, GULP1, U1, U6* | 1.21 |
|  |  | 79136645 - 80118728 | *GYPC, GLS, STAT1, STAT4* | 0.60 |
|  |  | 33371557 - 34245669 | *KCNH7* | 0.53 |
| **QM** | 3 | 115120882 - 116094718 | *AGAP1, SH3BP4* | 10.24 |
|  |  | 116281339 - 117476155 | *ACKR3, COPS8, IQCA1* | 1.11 |
|  |  | 16047644 - 17668165 | *ADAM15, DCST1, DCST2, ZBTB7B, FLAD1, CKS1B, PMVK, SHC1, KCNN3, ADAR, PBXIP1, CHRNB2, UBE2Q1, TDRD10, SHE, IL6R, ATP8B2, AQP10, HAX1, CFAP141, TPM3, NUP210L, RPS25, RAB13, JTB, CREB3L4, SLC39A1, CRTC2, DENND4B, GATAD2B, LENEP, U6, bta-mir-190b, UBAP2L, C3H1orf43, TPM3* | 0.85 |
| **QM** | 5 | 12392161 - 13042145 | *SOX5, CCDC59, METTL25, TMTC2, U6* | 1.30 |
| **QM** | 7 | 35599341 - 36667553 | *-* | 12.81 |
|  |  | 34712038 - 35535979 | *DTWD2, TNFAIP8, FAM170A, HSD17B4, DMXL1* | 1.43 |
|  |  | 36703665 - 37647123 | *-* | 0.65 |
| **QM** | 9 | 68407830 - 69493738 | *ARHGAP18, L3MBTL3, SAMD3, TMEM200A, TMEM244* | 3.03 |
| **QM** | 11 | 69281601 - 70267924 | *GALNT14, CAPN13, LCLAT1, LBH, YPEL5* | 1.14 |
|  |  | 67964661 - 69208404 | *ANXA4, GMCL1, MXD1, PCBP1, ANTXR1, GFPT1, NFU1, AAK1, ASPRV1* | 0.68 |
| **QM** | 13 | 42590562 - 43907865 | *ACSS1, GDI2, TASOR2, ASB13, VSX1, CST3, ENTPD6, SYNDIG1, PYGB, ABHD12, ANKRD16, CST11, MGC133636, CST8, CST7, APMAP, bta-mir-2285df* | 1.79 |
|  |  | 4908300 - 5892398 | *-* | 1.58 |
|  |  | 5893178 - 6614129 | *BTBD3, SNORA70* | 0.62 |
|  |  | 43948410 - 45175355 | *AKR1C4, AKR1C3, 20ALPHA-HSD, AKR1E2* | 0.55 |
| **QM** | 17 | 9152717 - 10109146 | *NR3C2, bta-mir-12037* | 16.14 |
|  |  | 10116634 - 11170670 | *EDNRA, ARHGAP10, PRMT9, TMEM184C, NR3C2, bta-mir-12037* | 8.18 |
|  |  | 11271022 - 11916089 | *TTC29, POU4F2, SLC10A7* | 0.71 |
| **QM** | 18 | 62142562 - 62848554 | *VSTM1, NLRP9, EPN1, U2AF2, CCDC106, ZNF581, ZNF784, ZNF865, ZNF524, FIZ1, ZNF579, SBK3, SBK2, SSC5D, ZNF628, C19orf85, ISOC2, SHISA7, UBE2S, PPP6R1, RPL28, TMEM238, TMEM190, IL11, COX6B2, PPP6R1, KMT5C, TMEM150B, BRSK1, HSPBP1, PTPRH, SYT5, TNNI3, TNNT1, PPP1R12C, EPS8L1, RDH13, GP6, NCR1, FCAR, KIR2DL5A, CACNG8, CACNG6, ZNF580, U6, bta-mir-7865, bta-mir-935, DNAAF3* | 0.92 |
| **QM** | 19 | 29253529 - 30229647 | *STX8, CFAP52, USP43, DHRS7C, GSG1L2, GLP2R, RCVRN, GAS7, MYH13, MYH8, MYH4, MYH1, MYH2, MYH3, SCO1, ADPRM, TMEM220* | 0.69 |
| **QM** | 22 | 44527914 - 46346946 | *ARHGEF3, TASOR, CCDC66, ERC2, bta-mir-2371* | 2.24 |
|  |  | 49820256 - 51668972 | *RBM5, RBM6, DCAF1, RBM15B, MANF, DOCK3, MAPKAPK3, CISH, HEMK1, C22H3orf18, CACNA2D2, TMEM115, CYB561D2, NPRL2, ZMYND10, RASSF1, TUSC2, HYAL2, HYAL1, IFRD2, SEMA3B, GNAI2, SLC38A3, GNAT1, SEMA3F, U6, HYAL3* | 2.04 |
|  |  | 42853528 - 44436705 | *CFAP20DC, FAM3D, FAM107A, ACOX2, KCTD6, PDHB, RPP14, HTD2, ABHD6, DNASE1L3, FLNB, SLMAP, U6, bta-mir-2370, PXK* | 0.73 |
| **A_1.125** | 1 | 131531825 - 131531825 | *DZIP1L, CLDN18, SOX14* | 0.53 |
| **A_1.125** | 2 | 6600270 - 6600270 | *MSTN, PMS1, OSGEPL1, ANKAR, ASNSD1, SLC40A1, WDR75, HIBCH, C2H2orf88, ORMDL1* | 0.80 |
|  |  | 125903194 - 125903194 | *PIGV, PPP1R8, STX12, FAM76A, SMPDL3B, RPA2, THEMIS2, KDF1, NUDC, NR0B2, IFI6, FGR, GPATCH3, GPN2, SFN, ZDHHC18, AHDC1, WASF2, GPR3, CD164L2, MAP3K6, SYTL1, TMEM222, WDTC1, SLC9A1, TENT5B, TRNP1, SCARNA1, U6* | 0.51 |
| **A_1.125** | 3 | 115120882 - 115120882 | *AGAP1, SH3BP4* | 0.76 |
| **A_1.125** | 5 | 7065478 - 7065478 | *NAV3, U6* | 0.76 |
| **A_1.125** | 7 | 34955707 - 34955707 | *DTWD2, TNFAIP8, DMXL1* | 1.50 |
| **A_1.125** | 9 | 68401422 - 68401422 | *ARHGAP18*  *L3MBTL3*  *SAMD3*  *TMEM200A*  *TMEM244* | 0.62 |
| **A_1.125** | 11 | 67964661 - 67964661 | *ANXA4, GMCL1, MXD1, PCBP1, ANTXR1, GFPT1, NFU1, AAK1, ASPRV1* | 0.87 |
| **A_1.125** | 17 | 8641625 - 8641625 | *IQCM* | 1.00 |
| **A_1.125** | 22 | 42593827 - 42593827 | *CFAP20DC, FAM3D, FAM107A, ACOX2, KCTD6, PDHB, RPP14, HTD2, ABHD6* | 0.56 |
| **A_1.125** | 30 | 40874754 - 40874754 | *-* | 0.56 |
| **A_1.2** | 1 | 131531825 - 132723340 | *DZIP1L, CLDN18, SOX14* | 0.60 |
| **A_1.2** | 2 | 6600270 - 7670949 | *MSTN, PMS1, OSGEPL1, ANKAR, ASNSD1, SLC40A1, WDR75, HIBCH, C2H2orf88, ORMDL1* | 1.01 |
|  |  | 58362479 - 58907364 | *SPOPL, NXPH2* | 0.59 |
|  |  | 125903194 - 126945896 | *PIGV, PPP1R8, STX12, FAM76A, SMPDL3B, RPA2, THEMIS2, KDF1, NUDC, NR0B2, IFI6, FGR, GPATCH3, GPN2, SFN, ZDHHC18, AHDC1, WASF2, GPR3, CD164L2, MAP3K6, SYTL1, TMEM222, WDTC1, SLC9A1, TENT5B, TRNP1, SCARNA1, U6* | 0.69 |
| **A_1.2** | 3 | 115120882 - 116094718 | *AGAP1, SH3BP4* | 1.04 |
| **A_1.2** | 5 | 7065478 - 8176883 | *NAV3, U6* | 0.80 |
|  |  | 12000970 - 12792909 | *CCDC59, METTL25, TMTC2* | 0.53 |
| **A_1.2** | 7 | 34955707 - 35897872 | *DTWD2, TNFAIP8, DMXL1* | 1.88 |
| **A_1.2** | 9 | 68401422 - 69476496 | *ARHGAP18, L3MBTL3, SAMD3, TMEM200A, TMEM244* | 0.75 |
| **A_1.2** | 11 | 67964661 - 69208404 | *ANXA4, GMCL1, MXD1, PCBP1, ANTXR1, GFPT1, NFU1, AAK1, ASPRV1* | 0.97 |
| **A_1.2** | 17 | 8641625 - 9641431 | *IQCM* | 1.26 |
| **A_1.2** | 22 | 42594928 - 44248276 | *CFAP20DC, FAM3D, FAM107A, ACOX2, KCTD6, PDHB, RPP14, HTD2, ABHD6, U6, PXK* | 0.58 |
|  |  | 48924379 - 49999285 | *RRP9, IQCF1, IQCF5, ALAS1, TEX264, IQCF2, IQCF6, GRM2, POC1A, RAD54L2, DCAF1, RBM15B, MANF, DOCK3, NISCH, TNNC1, SEMA3G, PHF7, BAP1, DNAH1, GLYCTK, DUSP7, RPL29, ACY1, ABHD14A, ABHD14B, PCBP4, GPR62, WDR82, PPM1M, TLR9, PARP3, MIRLET7G, bta-mir-135a-1, TWF2* | 0.54 |
| **A_1.2** | 30 | 40874754 - 43940345 | *-* | 0.54 |
| **A_1.5** | 1 | 131531825 - 132723340 | *DZIP1L, CLDN18, SOX14* | 0.85 |
| **A_1.5** | 2 | 6891780 - 7752741 | *COL5A2, COL3A1, PMS1, OSGEPL1, ANKAR, ASNSD1, SLC40A1, WDR75, bta-mir-2917, ORMDL1* | 2.70 |
|  |  | 33237706 - 34141020 | *FIGN, KCNH7* | 0.86 |
|  |  | 34152721 - 35362953 | *FIGN, KCNH7, KCNH7, GCA, IFIH1, FAP, GCG, DPP4, SLC4A10, U6* | 0.53 |
|  |  | 58362479 - 58907364 | *SPOPL, NXPH2* | 1.25 |
|  |  | 59144271 - 60080625 | *HNMT, THSD7B* | 1.55 |
|  |  | 60160111 - 61574417 | *THSD7B, U6* | 0.76 |
|  |  | 126497926 - 126976378 | *PDIK1L, PIGV, RPS6KA1, HMGN2, DHDDS, LIN28A, ARID1A, ZNF683, CRYBG2, CD52, UBXN11, SH3BGRL3, CEP85, CATSPER4, CNKSR1, ZNF593OS, FAM110D, ZNF593, C1orf232, KDF1, NUDC, NR0B2, GPATCH3, GPN2, SFN, ZDHHC18, WDTC1, SLC9A1, TENT5B, TRNP1* | 2.26 |
| **A_1.5** | 3 | 115120882 - 116094718 | *AGAP1, SH3BP4* | 4.04 |
| **A_1.5** | 5 | 7095612 - 8203831 | *-* | 0.67 |
|  |  | 12000970 - 12792909 | *CCDC59, METTL25, TMTC2, NAV3* | 0.70 |
| **A_1.5** | 7 | 35055453 - 36091735 | *DTWD2, TNFAIP8, DMXL1* | 4.64 |
|  |  | 36251748 - 37204273 | *-* | 0.99 |
| **A_1.5** | 9 | 12031049 - 13247101 | *RIMS1, KCNQ5* | 0.52 |
|  |  | 68407830 - 69493738 | *ARHGAP18, L3MBTL3, SAMD3, TMEM200A, TMEM244* | 2.16 |
| **A_1.5** | 11 | 67964661 - 69208404 | *ANXA4, GMCL1, MXD1, PCBP1, ANTXR1, GFPT1, NFU1, AAK1, ASPRV1* | 1.26 |
| **A_1.5** | 13 | 4908300 - 5892398 | *-* | 1.12 |
|  |  | 43067815 - 44592952 | *UCN3, ACSS1, GDI2, TASOR2, ASB13, VSX1, ENTPD6, NET1, TUBAL3, PYGB, ABHD12, ANKRD16, CST7, APMAP, CALML5, bta-mir-2285df, AKR1C4* | 0.64 |
| **A_1.5** | 17 | 9134022 - 10106791 | *NR3C2* | 3.10 |
|  |  | 10989562 - 11798681 | *EDNRA, TTC29, TMEM184C, U6* | 0.93 |
| **A_1.5** | 19 | 29222124 - 30214419 | *STX8, CFAP52, USP43, DHRS7C, GSG1L2, GLP2R, RCVRN, GAS7, MYH13, MYH8, MYH4, MYH1, MYH2, MYH3, SCO1, ADPRM, TMEM220* | 0.53 |
| **A_1.5** | 22 | 44527914 - 46346946 | *ARHGEF3, TASOR, CCDC66, ERC2, bta-mir-2371* | 0.87 |
|  |  | 49820256 - 51668972 | *RBM5, RBM6, DCAF1, RBM15B, MANF, DOCK3, MAPKAPK3, CISH, HEMK1, C22H3orf18, CACNA2D2, TMEM115, CYB561D2, NPRL2, ZMYND10, RASSF1, TUSC2, HYAL2, HYAL1, IFRD2, SEMA3B, GNAI2, SLC38A3, GNAT1, SEMA3F, U6, HYAL3* | 1.52 |
| Chr = chromosome; %Var = percentage of variance explained by the genomic window; UM = Unweighted method; QM = Quadratic method; A_1.125 = Non-Linear A method with a weighting factor equal to 1.125; A_1.2 = Non-Linear A method with a weighting factor equal to 1.2; A_1.5 = Non-Linear A method with a weighting factor equal to 1.5 | | | | |
