# Supplementary material for: Genome Wide Association Studies with Different Weighting Approaches Reveals Genomic Windows Associated with Meat Quality Traits in Beef Cattle
Source: Genes (Basel). 2026 Mar 28;17(4):385. doi: 10.3390/genes17040385 (PMC13116000; doi:10.3390/genes17040385)
Supplement: Supplementary file 1 [file genes-17-00385-s001.zip › Supplementary Materials/Supplementary_Table_S1.docx]

**Supplementary Table S1.** Genomic windows of 20 adjacent SNPs explaining 0.5% or more of the AGV, identified across five GWAS approaches for REA.

| Method | BTA | Genomic window | %Var |
| --- | --- | --- | --- |
| U | 2_75 | 75032049 - 75918514 | 0.80983 |
|  | 4_76 | 76581603 - 77774235 | 0.74832 |
|  | 4_80 | 80277910 - 81169085 | 0.51151 |
|  | 6_105 | 105912944 - 107227062 | 0.70784 |
|  | 12_30 | 30852113 - 32681456 | 0.77998 |
|  | 16_33 | 33655657 - 34881990 | 0.77685 |
|  | 19_19 | 19002409 - 19765067 | 0.80261 |
|  | 20_2 | 2522258 - 3588633 | 0.5212 |
|  | 22_42 | 42593389 - 44047775 | 0.84458 |
|  | 23_46 | 46249030 - 47505469 | 0.64797 |
| QM | 1_13 | 13778296 - 15074076 | 0.70832 |
|  | 2_75 | 75889261 - 77090209 | 1.34681 |
|  | 2_90 | 90237664 - 91563786 | 0.62723 |
|  | 3_23 | 23560725 - 24815752 | 1.95499 |
|  | 4_38 | 38363077 - 39786318 | 0.72404 |
|  | 4_80 | 80277910 - 81169085 | 1.3852 |
|  | 4_81 | 81743723 - 82869117 | 1.58771 |
|  | 6_105 | 105835255 - 107098263 | 2.08726 |
|  | 6_107 | 107226295 - 108591544 | 23.52949 |
|  | 6_108 | 108592440 - 109938102 | 1.61139 |
|  | 12_9 | 9026492 - 10122039 | 0.60877 |
|  | 12_32 | 32027380 - 33457798 | 11.83371 |
|  | 12_33 | 33476176 - 35054878 | 5.30147 |
|  | 16_34 | 34008163 - 35071667 | 9.71336 |
|  | 16_35 | 35137739 - 36067442 | 1.45828 |
|  | 18_42 | 42411072 - 43284189 | 0.64395 |
|  | 19_19 | 19002409 - 19765067 | 1.00137 |
|  | 19_19 | 19814327 - 20963068 | 11.13446 |
|  | 19_21 | 21018421 - 22039421 | 1.18217 |
|  | 22_43 | 43713828 - 45040991 | 1.49634 |
|  | 22_57 | 57498869 - 58165788 | 0.54534 |
|  | 23_46 | 46331376 - 47564094 | 4.59847 |
|  | 23_47 | 47625893 - 48714745 | 1.86822 |
|  | 27_15 | 15236071 - 16259785 | 0.82062 |
| A_1.125 | 2_75 | 75032049 - 75918514 | 0.85702 |
|  | 2_90 | 90182742 - 91367222 | 0.55041 |
|  | 4_76 | 76717317 - 77917876 | 0.79188 |
|  | 4_80 | 80277910 - 81169085 | 0.68843 |
|  | 5_52 | 52109863 - 53268394 | 0.51368 |
|  | 6_105 | 105912944 - 107227062 | 1.14013 |
|  | 12_30 | 30852113 - 32681456 | 1.10578 |
|  | 16_33 | 33980423 - 35056607 | 1.00792 |
|  | 19_19 | 19042090 - 19844499 | 0.89259 |
|  | 20_2 | 2522258 - 3588633 | 0.55288 |
|  | 22_42 | 42593389 - 44047775 | 1.07781 |
|  | 22_42 | 56522913 - 57676680 | 0.53006 |
|  | 23_46 | 46331376 - 47564094 | 0.90658 |
| A_1.2 | 2_75 | 75032049 - 75918514 | 0.86662 |
|  | 2_90 | 90182742 - 91367222 | 0.62869 |
|  | 4_76 | 76717317 - 77917876 | 0.79032 |
|  | 4_80 | 80277910 - 81169085 | 0.83678 |
|  | 5_52 | 52109863 - 53268394 | 0.58415 |
|  | 6_105 | 105912944 - 107227062 | 1.58094 |
|  | 6_107 | 107300290 - 108668355 | 0.75344 |
|  | 12_30 | 30852113 - 32681456 | 1.38714 |
|  | 12_32 | 32788874 - 34171532 | 0.59312 |
|  | 16_34 | 34008163 - 35071667 | 1.29952 |
|  | 19_19 | 19064643 - 19920352 | 1.00969 |
|  | 20_2 | 2522258 - 3588633 | 0.57722 |
|  | 22_42 | 42593827 - 44235949 | 1.26508 |
|  | 22_56 | 56522913 - 57676680 | 0.56752 |
|  | 23_46 | 46331376 - 47564094 | 1.15844 |
|  | 27_15 | 15236071 - 16259785 | 0.51271 |
| A_1.5 | 1_13 | 13778296 - 15074076 | 0.79773 |
|  | 1_69 | 69213596 - 70559726 | 1.06717 |
|  | 1_70 | 70569417 - 72055544 | 0.91805 |
|  | 1_98 | 98374868 - 99566422 | 0.57254 |
|  | 1_100 | 100156848 - 101291051 | 0.88327 |
|  | 2_75 | 75032049 - 75918514 | 0.69141 |
|  | 2_90 | 90237664 - 91563786 | 0.64003 |
|  | 3_22 | 22482395 - 23548978 | 0.5772 |
|  | 3_23 | 23560725 - 24815752 | 1.02755 |
|  | 4_38 | 38363077 - 39786318 | 0.82321 |
|  | 4_76 | 76717317 - 77917876 | 0.56552 |
|  | 4_80 | 80892637 - 82056167 | 1.64524 |
|  | 5_12 | 12704211 - 13494148 | 0.63763 |
|  | 5_52 | 52109863 - 53268394 | 0.75363 |
|  | 6_105 | 105912944 - 107227062 | 4.92636 |
|  | 6_107 | 107259582 - 108596491 | 3.05671 |
|  | 6_108 | 108899604 - 110262926 | 0.6816 |
|  | 12_8 | 8972547 - 10024033 | 1.62575 |
|  | 12_31 | 31814723 - 33308209 | 4.87489 |
|  | 12_33 | 33418450 - 34711526 | 0.95587 |
|  | 12_76 | 76661176 - 77943237 | 0.54652 |
|  | 16_34 | 34008163 - 35071667 | 2.65666 |
|  | 19_19 | 19002409 - 19765067 | 1.05131 |
|  | 19_19 | 19814327 - 20963068 | 2.03975 |
|  | 22_42 | 42594928 - 44248276 | 1.68288 |
|  | 22_44 | 44527914 - 46346946 | 2.11241 |
|  | 22_57 | 57498869 - 58165788 | 0.6717 |
|  | 23_46 | 46331376 - 47564094 | 2.04242 |
|  | 27_15 | 15236071 - 16259785 | 0.91434 |
|  | 28_39 | 39266326 - 40484243 | 0.5935 |
|  | 29_5 | 5784033 - 6869558 | 0.60476 |

BTA = position based on the genome of Bos taurus ARS-UCD2.0.114; Genomic window = SNPs present at the beginning and end of the genomic window; %Var = percentage of AGV explained by the genomic window. GWAS approaches: UM; QM = quadratic method; A_1.125 = non-linear A with weight 1.125; A_1.2 = non-linear A with weight 1.2; A_1.5 = non-linear A with weight 1.5.
