# Supplementary material for: Genome Wide Association Studies with Different Weighting Approaches Reveals Genomic Windows Associated with Meat Quality Traits in Beef Cattle
Source: Genes (Basel). 2026 Mar 28;17(4):385. doi: 10.3390/genes17040385 (PMC13116000; doi:10.3390/genes17040385)
Supplement: Supplementary file 1 [file genes-17-00385-s001.zip › Supplementary Materials/Supplementary_Table_S2.docx]

**Table S2.** Genomic windows of 20 adjacent SNPs explaining 0.5% or more of the AGV for SFT, identified across five GWAS approaches for SFT.

| Method | BTA | Genomic window | %Var |
| --- | --- | --- | --- |
| UM | 2_6 | 6600270 - 7670949 | 0.57553 |
|  | 3_115 | 115120882 - 116094718 | 0.51196 |
|  | 5_7 | 7065478 - 8176883 | 0.67706 |
|  | 7_34 | 34952996 - 35832365 | 1.10789 |
|  | 11_67 | 67964661 - 69208404 | 0.74574 |
|  | 17_8 | 8641625 - 9641431 | 0.70955 |
|  | 22_42 | 42593389 - 44047775 | 0.52058 |
|  | 30_40 | 40874754 - 43940345 | 0.57241 |
| QM | 1_131 | 131617330 - 133013900 | 1.54799 |
|  | 2_6 | 6902327 - 7807676 | 5.05112 |
|  | 2_7 | 7819857 - 9004095 | 1.21289 |
|  | 2_33 | 33371557 - 34245669 | 0.53152 |
|  | 2_58 | 58476871 - 59243122 | 1.38009 |
|  | 2_79 | 79136645 - 80118728 | 0.59957 |
|  | 2_126 | 126497926 - 126976378 | 1.81732 |
|  | 3_16 | 16047644 - 17668165 | 0.85484 |
|  | 3_115 | 115120882 - 116094718 | 10.24414 |
|  | 3_116 | 116281339 - 117476155 | 1.11367 |
|  | 5_12 | 12392161 - 13042145 | 1.30473 |
|  | 5_86 | 86160423 - 87212964 | 0.51512 |
|  | 7_34 | 34712038 - 35535979 | 1.43205 |
|  | 7_35 | 35599341 - 36667553 | 12.80575 |
|  | 7_36 | 36703665 - 37647123 | 0.64962 |
|  | 9_68 | 68407830 - 69493738 | 3.03335 |
|  | 11_67 | 67964661 - 69208404 | 0.67923 |
|  | 11_69 | 69281601 - 70267924 | 1.13725 |
|  | 13_4 | 4908300 - 5892398 | 1.58473 |
|  | 13_42 | 42590562 - 43907865 | 1.79318 |
|  | 13_43 | 43948410 - 45175355 | 0.55052 |
|  | 13_5 | 5893178 - 6614129 | 0.61667 |
|  | 17_10 | 10116634 - 11170670 | 8.1781 |
|  | 17_11 | 11271022 - 11916089 | 0.70685 |
|  | 17_9 | 9152717 - 10109146 | 16.13712 |
|  | 18_62 | 62142562 - 62848554 | 0.91847 |
|  | 19_29 | 29253529 - 30229647 | 0.69306 |
|  | 22_42 | 42853528 - 44436705 | 0.73085 |
|  | 22_44 | 44527914 - 46346946 | 2.24347 |
|  | 22_49 | 49820256 - 51668972 | 2.04406 |
| A_1.125 | 1_131 | 131531825 - 132723340 | 0.52529 |
|  | 2_6 | 6600270 - 7670949 | 0.79891 |
|  | 2_125 | 125903194 - 126945896 | 0.51372 |
|  | 3_115 | 115120882 - 116094718 | 0.7577 |
|  | 5_7 | 7065478 - 8176883 | 0.75693 |
|  | 7_34 | 34955707 - 35897872 | 1.50122 |
|  | 9_68 | 68401422 - 69476496 | 0.61628 |
|  | 11_67 | 67964661 - 69208404 | 0.86869 |
|  | 17_8 | 8641625 - 9641431 | 1.0014 |
|  | 22_42 | 42593827 - 44235949 | 0.55696 |
|  | 30_40 | 40874754 - 43940345 | 0.56463 |
| A_1.2 | 1_131 | 131531825 - 132723340 | 0.60238 |
|  | 2_6 | 6600270 - 7670949 | 1.00867 |
|  | 2_58 | 58362479 - 58907364 | 0.58907 |
|  | 2_125 | 125903194 - 126945896 | 0.68597 |
|  | 3_115 | 115120882 - 116094718 | 1.03673 |
|  | 5_7 | 7065478 - 8176883 | 0.79855 |
|  | 5_12 | 12000970 - 12792909 | 0.5303 |
|  | 7_34 | 34955707 - 35897872 | 1.87687 |
|  | 9_68 | 68401422 - 69476496 | 0.74938 |
|  | 11_67 | 67964661 - 69208404 | 0.97267 |
|  | 17_8 | 8641625 - 9641431 | 1.2604 |
|  | 22_42 | 42594928 - 44248276 | 0.58 |
|  | 22_48 | 48924379 - 49999285 | 0.53743 |
|  | 30_40 | 40874754 - 43940345 | 0.54285 |
| A_1.5 | 1_131 | 131531825 - 132723340 | 0.84918 |
|  | 2_6 | 6891780 - 7752741 | 2.69613 |
|  | 2_33 | 33237706 - 34141020 | 0.85768 |
|  | 2_34 | 34152721 - 35362953 | 0.53136 |
|  | 2_58 | 58362479 - 58907364 | 1.25157 |
|  | 2_59 | 59144271 - 60080625 | 1.55418 |
|  | 2_60 | 60160111 - 61574417 | 0.75541 |
|  | 2_126 | 126497926 - 126976378 | 2.26473 |
|  | 3_115 | 115120882 - 116094718 | 4.03639 |
|  | 5_7 | 7095612 - 8203831 | 0.66996 |
|  | 5_12 | 12000970 - 12792909 | 0.70277 |
|  | 7_35 | 35055453 - 36091735 | 4.64043 |
|  | 7_36 | 36251748 - 37204273 | 0.9927 |
|  | 9_12 | 12031049 - 13247101 | 0.5174 |
|  | 9_68 | 68407830 - 69493738 | 2.16058 |
|  | 11_67 | 67964661 - 69208404 | 1.26231 |
|  | 13_4 | 4908300 - 5892398 | 1.12129 |
|  | 13_43 | 43067815 - 44592952 | 0.64464 |
|  | 17_9 | 9134022 - 10106791 | 3.10484 |
|  | 17_10 | 10989562 - 11798681 | 0.92997 |
|  | 19_29 | 29222124 - 30214419 | 0.53198 |
|  | 22_44 | 44527914 - 46346946 | 0.87346 |
|  | 22_49 | 49820256 - 51668972 | 1.51869 |

BTA = position based on the genome of Bos taurus ARS-UCD2.0.114; Genomic window = SNPs present at the beginning and end of the genomic window; %Var = percentage of AGV explained by the genomic window. GWAS approaches: UM = unweighted method; QM = quadratic method; A_1.125 = non-linear A with weight 1.125; A_1.2 = non-linear A with weight 1.2; A_1.5 = non-linear A with weight 1.5.
